# Pentoxifylline and Norcantharidin Synergistically Suppress Melanoma Growth in Mice: A Multi-Modal In Vivo and In Silico Study

**DOI:** 10.3390/ijms26157522

**Published:** 2025-08-04

**Authors:** Israel Lara-Vega, Minerva Nájera-Martínez, Armando Vega-López

**Affiliations:** Laboratorio de Toxicología Ambiental, Escuela Nacional de Ciencias Biológicas, Instituto Politécnico Nacional, Av. Wilfrido Massieu s/n, Unidad Profesional Zacatenco, Mexico City 07738, Mexico; isralv@outlook.com (I.L.-V.);

**Keywords:** melanoma mouse model, pentoxifylline, norcantharidin, B16-F1, DBA/2J, RNA sequencing, ERBB2, combined treatment, PI3K/AKT/mTOR, melanogenesis

## Abstract

Melanoma is a highly aggressive skin cancer with limited therapeutic response. Targeting intracellular signaling pathways and promoting tumor cell differentiation are promising therapeutic strategies. Pentoxifylline (PTX) and norcantharidin (NCTD) have demonstrated antitumor properties, but their combined mechanisms of action in melanoma remain poorly understood. The effects of PTX (30 and 60 mg/kg) and NCTD (0.75 and 3 mg/kg), administered alone or in combination, in a DBA/2J murine B16-F1 melanoma model via intraperitoneal and intratumoral (IT) routes were evaluated. Tumor growth was monitored, and molecular analyses included RNA sequencing and immunofluorescence quantification of PI3K, AKT1, mTOR, ERBB2, BRAF, and MITF protein levels, and molecular docking simulations were performed. In the final stage of the experiment, combination therapy significantly reduced tumor volume compared to monotherapies, with the relative tumor volume decreasing from 18.1 ± 1.2 (SD) in the IT Control group to 0.6 ± 0.1 (SD) in the IT combination-treated group (n = 6 per group; *p* < 0.001). RNA-seq revealed over 3000 differentially expressed genes in intratumoral treatments, with enrichment in pathways related to oxidative stress, immune response, and translation regulation (KEGG and Reactome analyses). Minimal transcript-level changes were observed for BRAF and PI3K/AKT/mTOR genes; however, immunofluorescence showed reduced total and phosphorylated levels of PI3K, AKT1, mTOR, BRAF, and ERBB2. MITF protein levels and pigmentation increased, especially in PTX-treated groups, indicating enhanced melanocytic differentiation. Docking analyses predicted direct binding of both drugs to PI3K, AKT1, mTOR, and BRAF, with affinities ranging from −5.7 to −7.4 kcal/mol. The combination of PTX and NCTD suppresses melanoma progression through dual mechanisms: inhibition of PI3K/AKT/mTOR signaling and promotion of tumor cell differentiation.

## 1. Introduction

Melanoma represents one of the most aggressive and therapeutically challenging malignancies, characterized by rapid progression, high metastatic potential, and resistance to conventional treatments [1]. Despite significant advances in targeted therapies and immune checkpoint inhibitors, tumor heterogeneity and adaptive resistance mechanisms continue to pose major clinical obstacles [2], underscoring the urgent need for novel therapeutic strategies [3].

In this context, combination therapies have emerged as a promising approach to enhance treatment efficacy and overcome resistance by simultaneously targeting multiple oncogenic pathways. Recent preclinical studies have highlighted the potential of combining photodynamic and photothermal therapies, showing synergistic antitumor effects, particularly in murine models bearing B16 melanoma, although further studies are needed to assess long-term efficacy in metastatic settings [3]. Similarly, combinations involving radiotherapy or targeted radionuclide therapy have demonstrated significant tumor inhibition and systemic protection in mouse models, suggesting their potential as primary strategies for advanced melanoma [4]. Furthermore, immunotherapeutic approaches combining immune checkpoint inhibitors, such as anti-PD-1/L1 and anti-CTLA-4 antibodies, have shown enhanced tumor suppression in preclinical models, reinforcing the value of combined immunotherapies to improve clinical outcomes [5]. These findings support the rationale for developing multimodal strategies to address the challenges posed by resistance and metastasis in melanoma.

Among the compounds with potential therapeutic relevance, pentoxifylline (PTX), a methylxanthine derivative approved by the FDA for the treatment of peripheral arterial disease [6], has demonstrated well-documented immunomodulatory and anti-inflammatory properties [7,8]. Similarly, norcantharidin (NCTD), a demethylated analog of cantharidin, has shown anticancer activity across various tumor models, including melanoma [9,10].

Although both PTX and NCTD have been individually proposed as potential therapeutic agents against melanoma, the combined effects of these compounds on melanoma progression, particularly their impact on the expression and activation of key oncogenic signaling pathways such as PI3K/AKT, MAPK, BRAF, and mTOR, remain insufficiently understood [11,12]. Previous studies have reported that both PTX and NCTD modulate signaling pathways associated with tumor proliferation, apoptosis, and immune regulation, in both in vitro [12,13] and in vivo models [11,14]. However, the precise molecular mechanisms underlying these effects, as well as their potential synergistic actions, require further elucidation.

To address this knowledge gap, we employed an integrative systems biology approach, combining in vivo tumor growth monitoring, transcriptomic (RNA-seq) and proteomic (immunofluorescence) analyses to comprehensively characterize gene and protein expression changes following treatment. Additionally, molecular docking simulations were performed to predict potential interactions between PTX, NCTD, and key regulatory proteins involved in melanoma pathophysiology.

## 2. Results

### 2.1. Effects of Treatments on Tumor Growth

**Figure 1:**
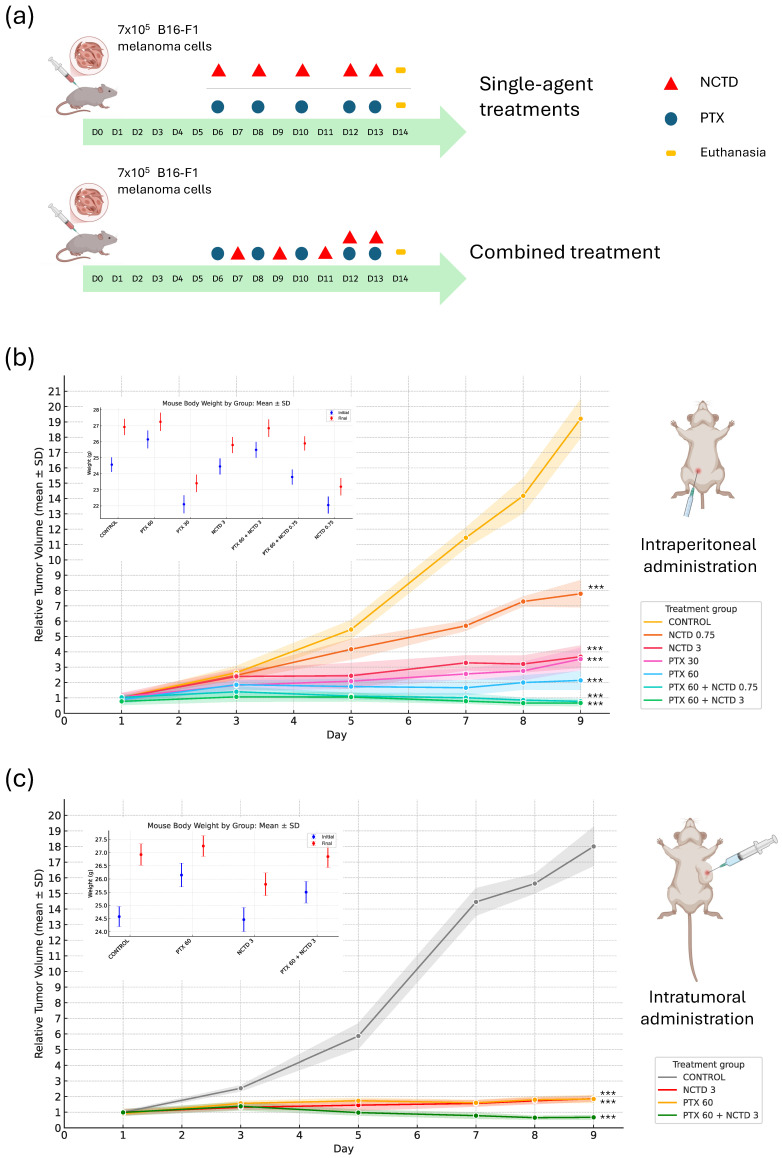
Tumor growth. (**a**) Schematic representation of the experimental timeline. On day 0 (D0), B16-F1 melanoma cells were subcutaneously injected into the right flank of mice. Treatments were administered starting on day 6 (D6), according to the indicated schedule. Mice were euthanized on day 14 (D14) for final analysis. (**b**) Relative tumor volume progression over time in seven experimental groups treated via intraperitoneal administration: Control, PTX 60 mg/kg, PTX 30 mg/kg, NCTD 3 mg/kg, PTX 60 mg/kg + NCTD 3 mg/kg, PTX 60 mg/kg + NCTD 0.75 mg/kg, and NCTD 0.75 mg/kg. (**c**) Relative tumor volume progression over time in four experimental groups treated via intratumoral administration: Control, PTX 60 mg/kg, NCTD 3 mg/kg, and PTX 60 mg/kg + NCTD 3 mg/kg. In both panels, each point represents the mean ± SD of six replicates per group. Comparisons were performed on D14. Additionally, body weight was monitored throughout the experiment for both administration routes as an indicator of general health and treatment-related toxicity. Statistical analysis was conducted using ANOVA followed by Tukey’s post hoc test. Asterisks indicate significant differences versus Control (*** *p* < 0.001).

To evaluate the antitumor efficacy of pentoxifylline (PTX), norcantharidin (NCTD), and their combination, we monitored tumor volume progression in B16-F1 melanoma-bearing DBA/2J mice following intraperitoneal and intratumoral administration schedules (Figure 1a). Tumor growth curves showed that all treatment groups exhibited reduced tumor volume compared to the Control group on day 14 (D14), with the most significant reductions observed in the combination treatment groups (Figure 1b,c). In the intraperitoneal administration series, the PTX 60 mg/kg + NCTD 3 mg/kg group displayed the greatest tumor suppression, followed by the PTX 60 mg/kg + NCTD 0.75 mg/kg group. Similarly, in the intratumoral administration series, the PTX 60 mg/kg + NCTD 3 mg/kg combination resulted in the most pronounced tumor growth inhibition.

Statistical analysis performed on D14 confirmed significant differences between treated groups and the Control group (ANOVA followed by Tukey’s post hoc test; *** *p* < 0.001). Furthermore, body weight was recorded throughout this study as a general indicator of animal health and potential treatment-related toxicity. The upper-left insets in Figure 1b,c show the body weight progression for each experimental group, revealing no significant weight loss in any treatment condition, suggesting that all treatment regimens were well tolerated under the experimental conditions.

A two-way ANOVA was performed to assess the effects of treatment, administration route, and their interaction on tumor volume on day 9 post-treatment (D14) (Supplementary Table S1). The analysis revealed a significant main effect of treatment (*p* < 0.001), indicating that tumor volume differed among treatment groups regardless of administration route. A significant main effect of administration route was also observed (*p* < 0.001), suggesting that the route of drug delivery (intraperitoneal vs. intratumoral) independently influenced tumor growth. Importantly, there was a significant interaction between treatment and route (*p* < 0.001), indicating that the effect of each treatment on tumor volume was dependent on the route of administration. Post hoc Tukey’s HSD test confirmed that the combination treatment (PTX 60 mg/kg + NCTD 3 mg/kg) administered intratumorally resulted in the most pronounced reduction in tumor volume compared to that in all other groups (*p* < 0.001). Additionally, for each individual treatment, intratumoral administration consistently showed greater tumor reduction than intraperitoneal administration. These findings highlight the enhanced efficacy of combination therapy and the superior antitumor effect of intratumoral delivery, supporting the observed macroscopic and molecular results.

**Figure 2:**
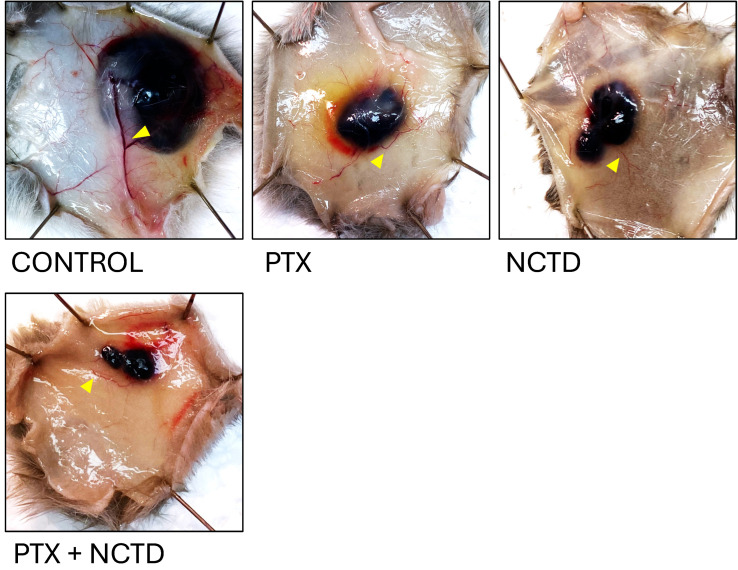
Representative photographs of tumors excised from melanoma-bearing mice (D14), illustrating tumor size differences across treatment groups on day 9 after treatment; treatments were administered intratumorally with n = 6 mice per group. The groups included Control (untreated), PTX (Pentoxifylline, 60 mg/kg), NCTD (Norcantharidin, 3 mg/kg), and PTX + NCTD (combined treatment at the same doses). Yellow arrowheads indicate main tumor-associated blood vessels.

Representative photographs of excised tumors on day 9 post-treatment (D14) are shown in Figure 2. Visual inspection clearly demonstrates differences in tumor size among the treatment groups. Tumors from the Control group appeared larger and more vascularized compared to those from the treated groups. Notably, the smallest tumors were observed in the combination group (PTX + NCTD), suggesting enhanced antitumor efficacy with the combined regimen. To highlight vascularization, yellow triangles were used to mark visible blood vessels supplying the tumors. In the Control group, larger and more prominent tumor-feeding vessels were observed, whereas treated groups, particularly the combination group, exhibited reduced vascularization. These macroscopic observations support the tumor growth inhibition results shown in Figure 1 and suggest a potential anti-angiogenic effect of the treatments. In Supplementary Figure S2, the representative images of excised tumors from melanoma-bearing mice following euthanasia can be observed.

### 2.2. Histological and Immunofluorescence Analysis of Tumor Tissues

#### 2.2.1. Regulation of MITF in Response to Treatments

**Figure 3:**
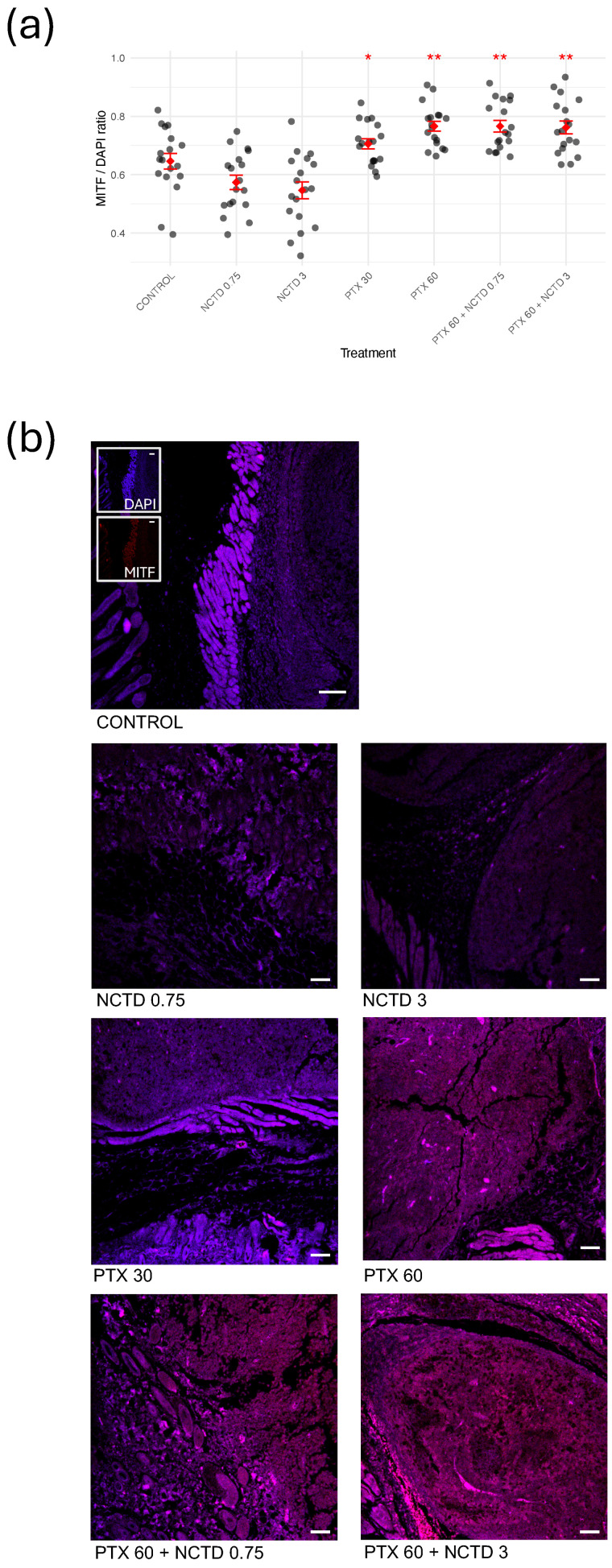
Regulation of MITF expression in melanoma tumors following treatment. (**a**) Quantification of MITF expression levels in tumor tissues by calculating the MITF/DAPI fluorescence ratio. Treatments with PTX 30 mg/kg, PTX 60 mg/kg, PTX 60 mg/kg + NCTD 0.75 mg/kg, and PTX 60 mg/kg + NCTD 3 mg/kg significantly increased MITF expression compared to that in the Control group (* *p* < 0.05; ** *p* < 0.01). (**b**) Representative immunofluorescence merge images at 10× magnification showing MITF staining (magenta/pink signal) in tumor sections from each treatment group. For each sample, three regions of interest were analyzed per tumor section, resulting in 18 data points per group (n = 6 mice). Nuclei were counterstained with DAPI (blue). White scale bars represent 100 µm in all images.

To investigate the effect of treatments on melanocytic differentiation, we evaluated the expression of microphthalmia-associated transcription factor (MITF) in tumor tissues of intraperitoneal groups by immunofluorescence analysis (Figure 3). Quantitative analysis of the MITF/DAPI fluorescence ratio revealed a significant increase in MITF expression in tumors from mice treated with PTX 30 mg/kg, PTX 60 mg/kg, PTX 60 mg/kg + NCTD 0.75 mg/kg, and PTX 60 mg/kg + NCTD 3 mg/kg compared to the Control group (Figure 3a). Among these, the PTX 60 mg/kg-treated groups showed the most pronounced increase, suggesting that pentoxifylline plays a key role in modulating MITF levels. Representative immunofluorescence images (Figure 3b) further support these findings. Tumor sections from PTX-treated groups exhibited a stronger fluorescence signal, corresponding to MITF expression. This enhanced staining was most prominent in the combination group (PTX 60 mg/kg + NCTD 3 mg/kg), corroborating the quantitative data and suggesting that both monotherapy and combination therapy promote MITF upregulation in melanoma tissues.

#### 2.2.2. Expression and Activation of Key Oncogenic Signaling Proteins

**Figure 4:**
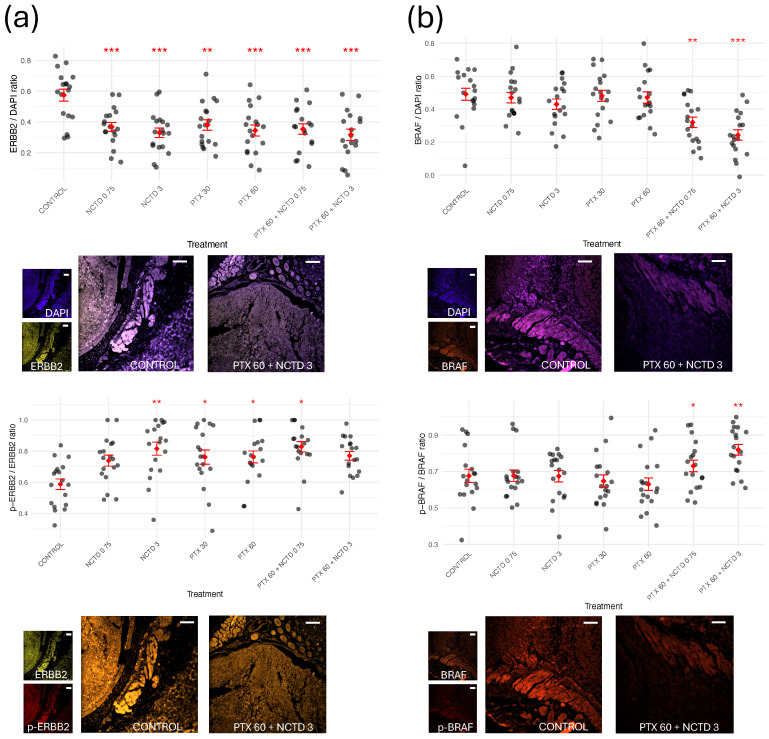
Effects of treatments on the expression and activation of ERBB2 and BRAF in melanoma tumors. Quantification of total protein expression (protein/DAPI ratio, upper graphs in each panel) and phosphorylation status (phosphorylated protein/total protein ratio, lower graphs in each panel) for (**a**) ERBB2 and (**b**) BRAF. Representative immunofluorescence images at 10× magnification (located below each corresponding ratio graph) illustrate protein localization and relative fluorescence intensity in tumor sections from the Control group and combined treatment (PTX 60 mg/kg + NCTD 3 mg/kg). For each sample, three regions of interest were analyzed per tumor section, resulting in 18 data points per group (n = 6 mice). Results are presented as mean ± SD. Statistical analysis was performed using ANOVA followed by Tukey’s post hoc test. Asterisks indicate significant differences versus the Control group (* *p* < 0.05; ** *p* < 0.01; *** *p* < 0.001). Nuclei were counterstained with DAPI (blue). White scale bars represent 100 µm in all images.

**Figure 5:**
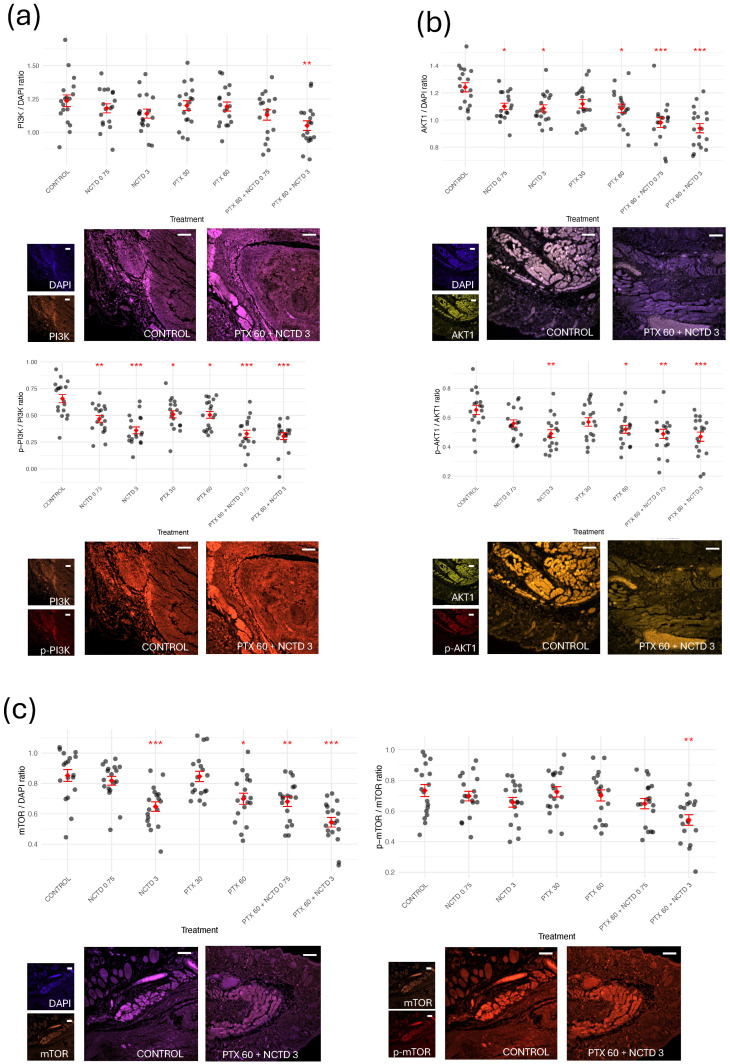
Effects of treatments on the expression and activation of PI3K, AKT1, and mTOR in melanoma tumors. Quantification of total protein expression (protein/DAPI ratio, upper/left graphs in each panel) and phosphorylation status (phosphorylated protein/total protein ratio, lower/right graphs in each panel) for (**a**) PI3K, (**b**) AKT1, and (**c**) mTOR. Representative immunofluorescence images at 10× magnification (located below each corresponding ratio graph) illustrate protein localization and relative fluorescence intensity in tumor sections from the Control group and combined treatment (PTX 60 mg/kg + NCTD 3 mg/kg). For each sample, three regions of interest were analyzed per tumor section, resulting in 18 data points per group (n = 6 mice). Results are presented as mean ± SD. Statistical analysis was performed using ANOVA followed by Tukey’s post hoc test. Asterisks indicate significant differences versus the Control group (* *p* < 0.05; ** *p* < 0.01; *** *p* < 0.001). Nuclei were counterstained with DAPI (blue). White scale bars represent 100 µm in all images.

To investigate the impact of the treatments on key signaling pathways involved in melanoma progression, we evaluated the expression and activation status of ERBB2, BRAF, PI3K, AKT1, and mTOR by immunofluorescence analysis (Figure 4 and Figure 5). For each protein, both total expression (protein/DAPI ratio) and activation level (phosphorylated protein/total protein ratio) were quantified. In the case of ERBB2 (Figure 4a), all treatment groups showed a significant reduction in total ERBB2 expression compared to the Control group, with the most pronounced decrease observed in the PTX 60 mg/kg + NCTD 3 mg/kg combination group. Notably, the phosphorylation status of ERBB2 (p-ERBB2/ERBB2 ratio) exhibited a divergent response: ERBB2 phosphorylation significantly increased in the monotherapy groups (PTX and NCTD), whereas only the combination therapy led to a significant reduction in ERBB2 phosphorylation. This paradoxical increase in phosphorylation despite reduced total ERBB2 levels in the monotherapy groups may reflect a compensatory feedback activation, a phenomenon previously described in other tumor models exposed to targeted therapies [15,16]. For BRAF (Figure 4b), a significant decrease in total BRAF expression was observed in the combination groups. Interestingly, phosphorylated BRAF (p-BRAF/BRAF ratio) was significantly increased in the combination groups, suggesting a potential compensatory activation of BRAF in response to upstream pathway inhibition. PI3K levels (Figure 5a) showed a marked reduction in total protein expression in the combination group, while the p-PI3K/PI3K ratio was significantly reduced across all treated groups, indicating effective downregulation of the PI3K pathway. Regarding AKT1 (Figure 5b), total AKT1 expression was significantly decreased in all treatment groups except PTX 30 mg/kg, while phosphorylated AKT1 levels (p-AKT1/AKT1 ratio) were significantly lower in the NCTD 3 mg/kg, PTX 60 mg/kg, and combination groups compared to the Control group. Finally, mTOR analysis (Figure 5c) revealed a significant reduction in both total mTOR expression and its phosphorylation status in the combination groups, with the most pronounced effect observed in the PTX 60 mg/kg + NCTD 3 mg/kg group. Representative immunofluorescence images for each protein are shown in the lower panel of each graph, highlighting the differences in fluorescence intensity between the Control and treated groups. These results indicate that PTX and NCTD treatments, particularly in combination, effectively downregulate multiple key oncogenic pathways in melanoma tumors, including the PI3K/AKT/mTOR and ERBB2/BRAF signaling axes.

### 2.3. Transcriptomic Analysis via RNA-Seq

#### 2.3.1. Differential Gene Expression Analysis

**Figure 6:**
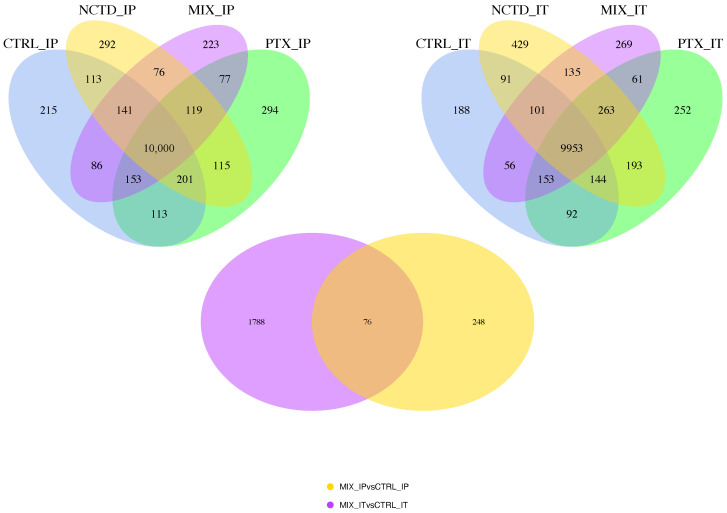
Differentially expressed genes (DEGs) identified by RNA-seq analysis across treatments and administration routes. Venn diagrams showing the number of DEGs in tumor tissues from mice treated via intraperitoneal (**left**) or intratumoral (**right**) routes, compared to their respective Controls (CONTROL_IP and CONTROL_IT). Each treatment group includes PTX, NCTD, and MIX (PTX + NCTD). The lower panel shows the overlap of DEGs between MIX_IP vs. CONTROL_IP and MIX_IT vs. CONTROL_IT comparisons, highlighting commonly regulated genes by the combination therapy regardless of the administration route.

To explore the transcriptomic changes induced by the treatments, differential gene expression analysis was performed by comparing each treatment group with its respective control for both administration routes (intraperitoneal and intratumoral). Figure 6 shows Venn diagrams illustrating the number of differentially expressed genes (DEGs) identified across the experimental conditions. For the intraperitoneal administration (IP), a total of 10,024 genes were commonly detected as differentially expressed across all four groups (CONTROL_IP, PTX_IP, NCTD_IP, and MIX_IP), while additional unique and shared DEGs were identified for each treatment comparison (Figure 6, left panel). Similarly, for the intratumoral administration (IT), 9953 DEGs were commonly detected across the CONTROL_IT, PTX_IT, NCTD_IT, and MIX_IT groups, with distinct subsets of DEGs specific to each treatment condition (Figure 6, right panel). Additionally, a Venn diagram comparing DEGs from the MIX_IP vs. CONTROL_IP and MIX_IT vs. CONTROL_IT contrasts reveals a subset of 76 DEGs commonly regulated by the combination treatment regardless of the administration route (Figure 6, bottom panel). This overlap suggests the existence of core transcriptomic responses to the combined therapy, independent of the delivery method.

**Figure 7:**
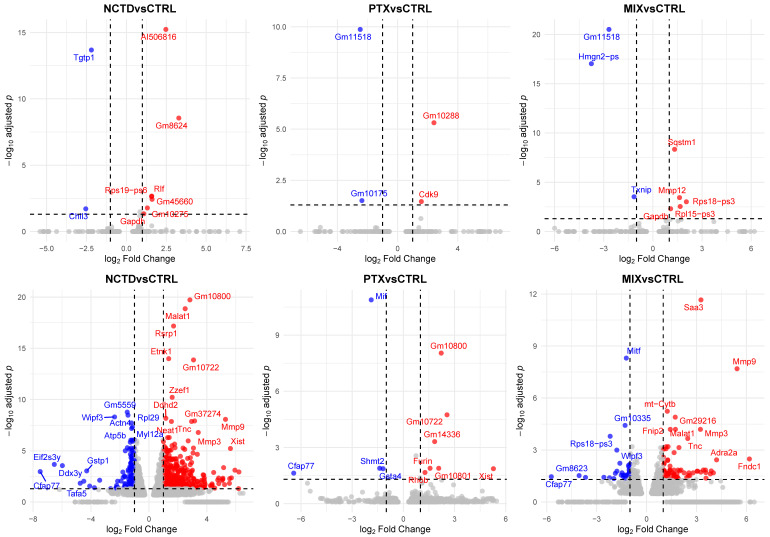
Volcano plots illustrating differential gene expression profiles for each treatment versus control. Upper plots: intraperitoneal administration groups: NCTD_IP vs. CONTROL_IP, PTX_IP vs. CONTROL_IP, and MIX_IP vs. CONTROL_IP. Lower plots: intratumoral administration groups: NCTD_IT vs. CONTROL_IT, PTX_IT vs. CONTROL_IT, and MIX_IT vs. CONTROL_IT. Each plot displays log_2_ fold change (x-axis) versus −log_10_-adjusted *p*-value (y-axis). Red dots represent significantly upregulated genes, blue dots indicate significantly downregulated genes (adjusted *p* < 0.05, |log_2_FC| ≥ 1), and gray dots represent non-significant genes. In each plot are labeled the representative differentially expressed genes to facilitate visualization.

To further explore the transcriptomic alterations induced by each treatment, volcano plots were generated for all pairwise comparisons against their respective Control groups (Figure 7). Each plot displays the distribution of DEGs, with the x-axis representing log_2_ fold change (log_2_FC) and the y-axis representing the −log_10_-adjusted *p*-value (padj). For the intraperitoneal administration groups (Figure 7, upper panels), treatments with PTX, NCTD, and the combination (MIX) resulted in a progressively increasing number of DEGs when compared to CONTROL_IP. Specifically, NCTD induced 288 DEGs, PTX induced 243 DEGs, and MIX induced 324 DEGs (Supplementary Figure S3), indicating a modest but notable increase in the transcriptomic response with combination therapy. In contrast, the intratumoral administration groups (Figure 7, lower panels) exhibited a much higher DEG count across all treatments when compared to CONTROL_IT. NCTD_IT generated 3233 DEGs, PTX_IT resulted in 1070 DEGs, and the combination MIX_IT induced 1864 DEGs (Supplementary Figure S3). These results indicate that intratumoral delivery, particularly NCTD monotherapy, elicited a broader transcriptional response compared to intraperitoneal administration, with MIX_IT also showing a substantial number of DEGs. Overall, these findings suggest that both the administration route and treatment type strongly influence the extent of transcriptomic modulation in melanoma tumors, with intratumoral treatments resulting in a significantly higher number of differentially expressed genes than intraperitoneal treatments.

#### 2.3.2. Clustered Gene Expression Patterns

**Figure 8:**
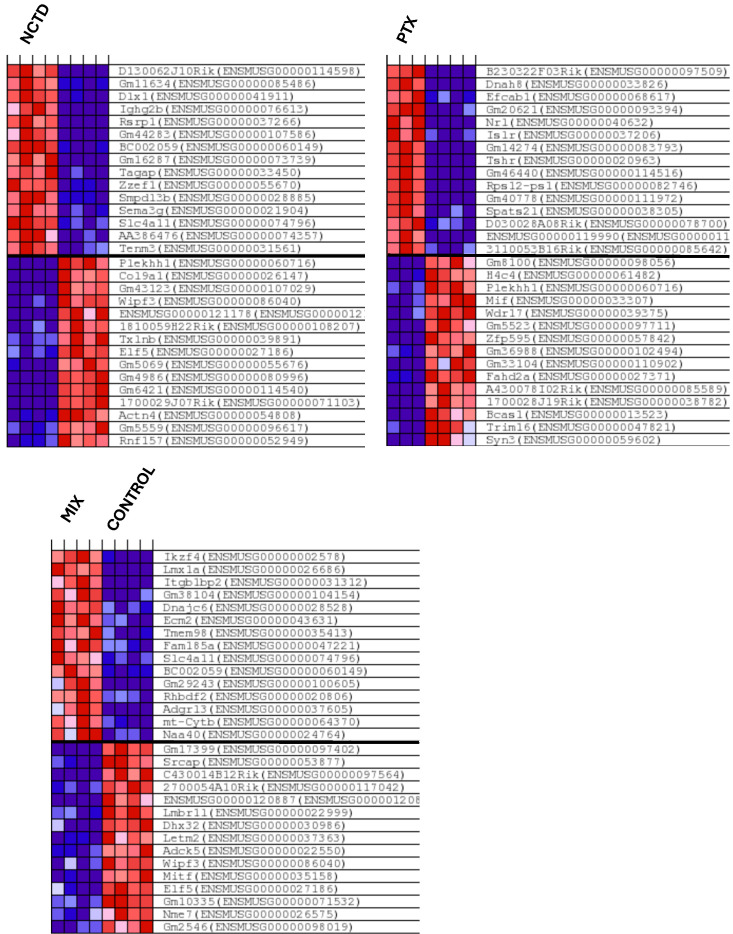
Hierarchical clustering and heatmap visualization of DEGs in intratumoral treatment groups. Heatmaps from GSEA-GO enrichment analyses display the top 15 features for each phenotype based on read count data. Gene expression profiles are shown for NCTD_IT vs. CONTROL_IT, PTX_IT vs. CONTROL_IT, and MIX_IT vs. CONTROL_IT. Each row represents a single gene, and each column corresponds to a tumor sample (n = 3–4 per group). The color scale indicates relative gene expression levels, with red representing upregulation and blue indicating downregulation.

To explore global transcriptional changes induced by the treatments, hierarchical clustering and heatmap visualization were performed using the DEGs from GO enrichment analyses from each comparison versus the Controls. For the intratumoral administration groups (Figure 8), distinct gene expression profiles are observed for each treatment (NCTD_IT, PTX_IT, and MIX_IT) compared to CONTROL_IT. The heatmaps reveals clear separation between Control and treated samples, with the NCTD_IT and MIX_IT groups showing the most pronounced gene expression shifts. These results are consistent with the higher number of DEGs identified in the intratumoral comparisons. For the intraperitoneal administration groups, hierarchical clustering patterns are presented in Supplementary Figure S4, showing less pronounced but still detectable transcriptional differences compared to CONTROL_IP. In Supplementary Figure S5 are also presented the intratumoral administration groups and hierarchical clustering patterns for the top 50 features. Overall, these clustered expression patterns indicate that both the route of administration and the treatment type strongly influence the global gene expression landscape in melanoma tumors, with intratumoral treatments inducing more robust transcriptional changes than intraperitoneal treatments.

#### 2.3.3. Functional Enrichment and Pathway Analysis

**Figure 9:**
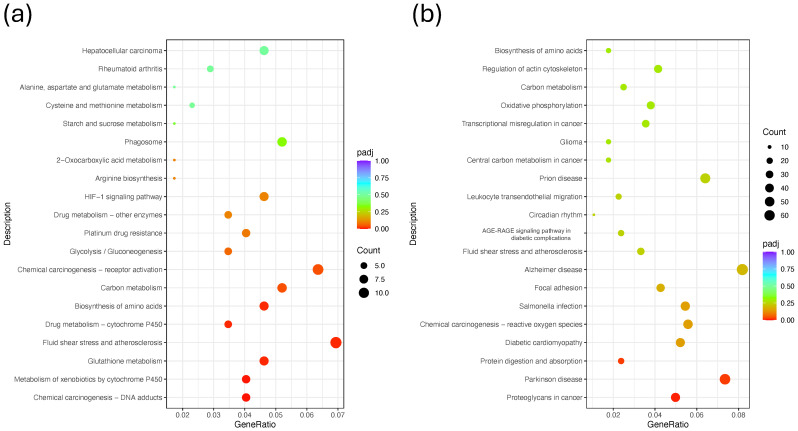
KEGG pathway enrichment analysis of DEGs induced by treatments. (**a**) Enriched pathways for intraperitoneal administration groups (MIX_IP vs. CONTROL_IP). (**b**) Enriched pathways for intratumoral administration groups (MIX_IT vs. CONTROL_IT). Dot plots display the most significantly enriched KEGG pathways, with dot size indicating the number of DEGs associated with each pathway and color representing the adjusted *p*-value (padj). Pathways are ranked by statistical significance.

To gain insight into the biological processes and pathways affected by the treatments, KEGG pathway enrichment analysis was performed using the DEGs from each comparison against the respective Control group. For the intraperitoneal administration groups (Figure 9a and Figure S6a), enriched pathways included oxidative phosphorylation, chemical carcinogenesis, reactive oxygen species, melanoma, carbon metabolism, and biosynthesis of amino acids. These pathways suggest that the treatments modulated processes related to energy metabolism, oxidative stress response, and cancer-related signaling. For the intratumoral administration groups (Figure 9a and Figure S6a), the enriched pathways were more diverse and numerous, reflecting the higher DEG count observed in these groups. Significant pathways included NF-kappa B signaling, the HIF-1 signaling pathway, melanoma, focal adhesion, leukocyte transendothelial migration, oxidative phosphorylation, and circadian rhythm, among others. Notably, several cancer-associated and immune-related pathways were overrepresented, particularly in the MIX_IT and NCTD_IT groups. These findings indicate that intratumoral treatments, especially with NCTD and the combination therapy, elicited broader and more diverse biological pathway alterations compared to intraperitoneal treatments, reinforcing the stronger transcriptomic impact observed in previous analyses.

**Figure 10:**
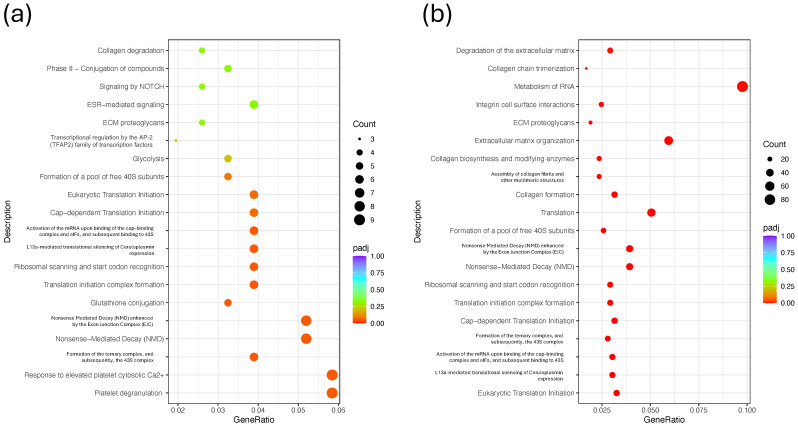
Reactome pathway enrichment analysis of DEGs induced by treatments. (**a**) Enriched pathways for intraperitoneal administration groups (MIX_IP vs. CONTROL_IP). (**b**) Enriched pathways for intratumoral administration groups (MIX_IT vs. CONTROL_IT). Dot plots display the most significantly enriched Reactome pathways, with dot size indicating the number of DEGs associated with each pathway and color representing the adjusted *p*-value (padj). Pathways are ranked by statistical significance.

To further characterize the biological functions affected by the treatments, Reactome pathway enrichment analysis was performed, comparing the DEGs from each treatment versus the Control. For the intraperitoneal treatment groups (Figure 10a and Figure S7a), the most significantly enriched pathways included “Respiratory electron transport and ATP synthesis”, “TP53 regulates metabolic genes”, “Formation of ATP by chemiosmotic coupling”, “Mitochondrial biogenesis”, “Glycolysis”, and “mTOR signaling”, among others. These pathways suggest that the treatments modulate mitochondrial activity, metabolic processes, and key signaling cascades such as the PI3K-mTOR axis. In the intratumoral treatment groups (Figure 10a and Figure S7a), a broader set of pathways were enriched, reflecting the higher DEG counts observed previously. Notable pathways included “Eukaryotic translation initiation”, “Nonsense-mediated decay (NMD)”, “Cap-dependent translation initiation”, “Glutathione conjugation”, “TNF receptor signaling”, “ECM proteoglycans”, and “Extracellular matrix organization”. These results highlight the strong modulation of translational control, stress responses, and extracellular matrix remodeling in response to intratumoral drug administration. Overall, these findings suggest that intratumoral treatments, especially with NCTD and combination therapy, induce broader alterations in translation regulation, stress response, and tumor microenvironment remodeling, whereas intraperitoneal treatments predominantly affect energy metabolism and mitochondrial function.

### 2.4. In Silico Analysis of Drug–Target Interactions

To predict the potential molecular targets of pentoxifylline (PTX) and norcantharidin (NCTD), molecular docking analyses were performed using key proteins involved in melanoma progression, oncogenic signaling, angiogenesis, and the tumor microenvironment. The results, summarized in Table 1, reveal that both PTX and NCTD showed binding affinities (ΔG values ranging from −3.5 to −7.8 kcal/mol) toward several oncogenic signaling proteins, transcription factors, and extracellular matrix regulators. Notably, PTX exhibited stronger binding energies than NCTD in the majority of the evaluated targets, especially for CD117 (−7.4 kcal/mol), B-RAF (−7.4 kcal/mol), PTGS2 (COX-2, −7.3 kcal/mol), and MMP-9 (−7.0 kcal/mol).

Importantly, several binding residues identified for PTX and NCTD are located in functionally critical regions of the target proteins. For example,

- For B-RAF, PTX showed predicted interactions with Lys483 and Asp594, both essential residues within the ATP binding site [17], suggesting potential interference with kinase activity;
- For mTOR, PTX docked at residues Gln1901, His2410, and Asp2412, located near the kinase catalytic site [18];
- For PIK3CA (PI3K catalytic subunit alpha), PTX interacted with residues such as His670 and Arg818, known to participate in PI3K activation [19];
- For HIF1A, NCTD showed the strongest binding energy (−7.8 kcal/mol), with interactions at residues Arg383 and His374, both involved in transcriptional regulation under hypoxic conditions [20].

Similarly, PTX showed notable binding to the NF-κB p50 subunit at Lys147, a residue implicated in DNA binding and transcriptional activation [21,22]. These in silico findings suggest that both drugs may potentially influence tumor-related processes by interacting with multiple molecular targets, including signaling kinases (e.g., PI3K, AKT1, mTOR, B-RAF), transcription factors (MITF, HIF1A, NF-κB), and proteins involved in tumor microenvironment remodeling (MMPs, VEGF-A, CD117).

**Figure 11:**
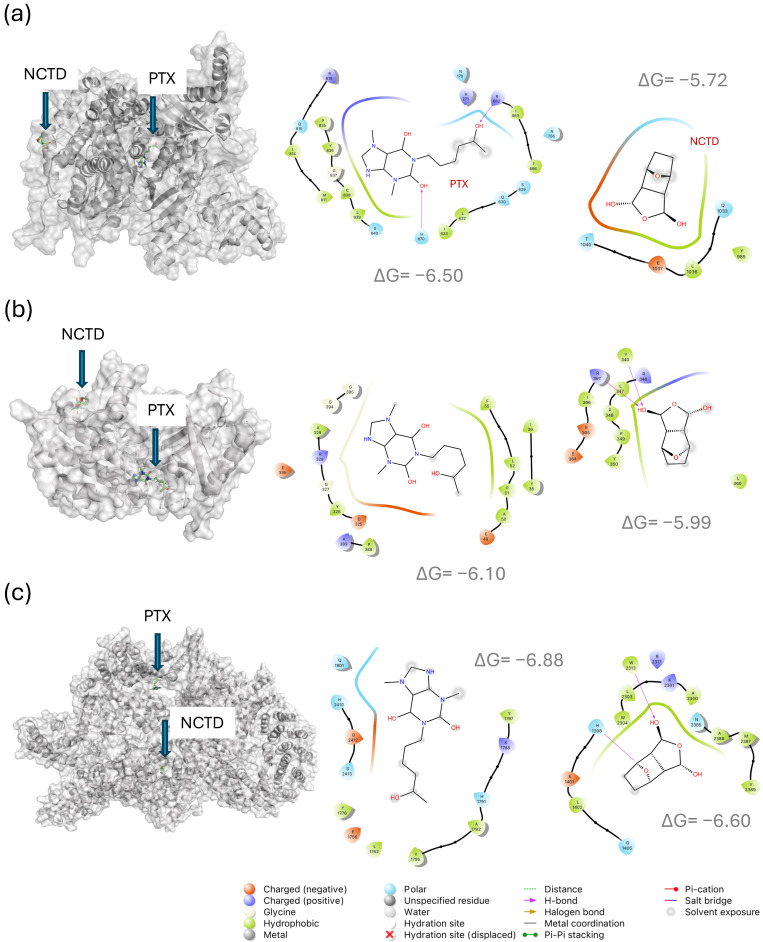
Representative molecular docking poses of pentoxifylline (PTX) and norcantharidin (NCTD) with key proteins in the PI3K/AKT/mTOR signaling pathway. (**a**) PI3K (PDB ID: 4A55), (**b**) AKT1 (PDB ID: 3O96), and (**c**) mTOR (PDB ID: 6BCX). For each target, the 3D binding pose of NCTD and PTX is shown (left panels), along with corresponding 2D interaction maps (right panels) detailing key amino acid residues involved in ligand binding. Binding energies (ΔG, kcal/mol) are indicated for each interaction.

To illustrate the predicted binding modes and molecular interactions, docking poses of PTX and NCTD with three representative signaling proteins—PI3K (PDB ID: 4A55), AKT1 (PDB ID: 3O96), and mTOR (PDB ID: 6BCX)—were visualized in both three-dimensional (3D) and two-dimensional (2D) formats (Figure 11). For PI3K (Figure 11a), PTX exhibited a predicted binding energy of −6.50 kcal/mol, interacting with residues such as His670, Arg662, and Arg818, located near the ATP-binding region. NCTD showed a slightly lower binding energy (−5.72 kcal/mol), forming contacts with Gln1033, Leu1036, and Glu1037, situated close to the catalytic site. For AKT1 (Figure 11b), PTX showed a binding energy of −6.10 kcal/mol, interacting with Leu52, Pro51, and Asp325, while NCTD exhibited a binding energy of −5.99 kcal/mol, engaging residues such as Leu360, Tyr340, and Arg367, all located near functionally relevant regions. In the case of mTOR (Figure 11c), PTX displayed a predicted binding energy of −6.88 kcal/mol, contacting residues Gln1901, His2410, and Asp2412, while NCTD showed −6.60 kcal/mol, interacting with His1398, Trp2313, and Trp2304. The 2D diagrams illustrate hydrogen bonds, hydrophobic contacts, and electrostatic interactions. These molecular docking results suggest that both compounds may interact with regulatory regions of PI3K/AKT/mTOR components, influencing their signaling activity.

To validate the molecular docking protocol employed in this study, we included four well-characterized inhibitors as positive Controls for each of the key melanoma-related targets (Supplementary Table S2). The binding affinities obtained were consistent with their known biological activity: Vemurafenib (PLX4032) for BRAF (PubChem ID: 42611257) showed a docking score of −10.5 kcal/mol; MK-2206 for AKT1 (PubChem ID: 24964624) scored −7.7 kcal/mol; Rapamycin (Sirolimus) for mTOR (PubChem ID: 5284616) yielded −9.8 kcal/mol; and Lapatinib for ERBB2 (PubChem ID: 208908) showed −8.3 kcal/mol. These values reflect the strong binding expected for established inhibitors and support the reliability of our docking methodology.

Overall, the combined in vivo, transcriptomic, molecular, and in silico analyses indicate that PTX and NCTD, both individually and in combination, modulate key signaling pathways involved in melanoma progression, proliferation, and differentiation. These multimodal effects were more evident with the combination treatment, underscoring the potential benefit of simultaneously targeting multiple regulatory pathways. A detailed integrative discussion of these results is provided in the following section.

## 3. Discussion

In this study, we employed a well-established preclinical model to investigate the antitumor effects of pentoxifylline (PTX) and norcantharidin (NCTD) in melanoma. Specifically, we used DBA/2J mice, a strain characterized by mutations in the Tyrp1 and Gpnmb genes that impair melanosome function, leading to pigment dispersion in the iris and contributing to the development of pigmentary glaucoma. These pigmentation characteristics make DBA/2J mice a valuable model for studying pigmentation-related diseases, including melanoma [23]. Additionally, its light-colored coat facilitates the macroscopic and histological assessment of pigmented tumors, such as those derived from the B16-F1 melanoma cell line. The DBA/2J strain has also been previously reported to provide favorable tumor take rates and reproducible growth kinetics for subcutaneous Cloudman S91 melanoma implantation, making it a suitable choice for evaluating tumor progression and therapeutic interventions with B16-F1 melanoma [24]. We selected the B16-F1 cell line due to its high tumorigenicity and its well-documented use in syngeneic mouse models for studying melanoma biology and therapy response [25]. This cell line allowed for the evaluation of both tumor growth dynamics and molecular changes within the tumor microenvironment. Furthermore, by including both intraperitoneal and intratumoral routes of drug administration, we were able to compare systemic versus local delivery strategies. The intraperitoneal route provided insights into systemic drug effects, while the intratumoral administration allowed for direct tumor-targeted delivery, potentially minimizing off-target toxicity and enhancing local drug concentration [26].

Treatment with PTX, NCTD, and their combination resulted in significant tumor volume reduction in B16-F1 melanoma-bearing DBA/2J mice. Both administration routes were effective in decreasing tumor growth; however, intratumoral administration, particularly the combination of PTX and NCTD, produced the most pronounced antitumor effects. These findings suggest that local delivery enhances drug bioavailability at the tumor site, potentially increasing therapeutic efficacy while reducing systemic exposure [3]. The differences in the number of DEGs observed between intraperitoneal and intratumoral administration (Supplementary Figure S3) may reflect underlying pharmacokinetic and tissue distribution differences associated with each route. Intratumoral injection delivers the drug directly into the tumor microenvironment, achieving higher local drug concentrations and bypassing systemic metabolism and distribution barriers that often limit drug availability at the tumor site when administered intraperitoneally or intravenously [27,28]. This localized exposure likely enhances cellular uptake and engagement with molecular targets within the tumor, thereby amplifying transcriptional responses. In contrast, drugs administered via the intraperitoneal route undergo systemic absorption, dilution, and hepatic metabolism, which can reduce the effective concentration reaching the tumor tissue [29]. Moreover, studies in murine models have shown that intratumoral delivery results in superior retention within the tumor and increased pharmacodynamic effects compared to systemic routes [3,30]. These pharmacokinetic considerations may explain the stronger transcriptomic modulation observed following intratumoral treatments in our model. The superior efficacy observed with the combination treatment may be attributed to the synergistic modulation of multiple oncogenic pathways, as both PTX and NCTD target distinct but complementary molecular mechanisms involved in melanoma progression [11]. Additionally, the lack of significant body weight loss across treatment groups indicates a favorable toxicity profile under the tested conditions.

One of the most notable molecular findings in this study was the upregulation of MITF expression in tumors from PTX-treated groups, as confirmed by immunofluorescence quantification. MITF is a key transcription factor involved in melanocyte differentiation and melanogenesis, and its expression is tightly regulated by upstream signaling pathways such as MAPK/ERK and PI3K/AKT [31]. The increase in MITF levels, especially in PTX monotherapy and combination groups, was accompanied by macroscopic evidence of enhanced pigmentation in the tumors, suggesting a shift towards a more differentiated melanoma phenotype. This observation is consistent with previous studies reporting that tumor cell differentiation, characterized by increased MITF and melanin production, can be associated with reduced proliferation and aggressiveness in melanoma [32]. Our results suggest that PTX may promote tumor cell differentiation through modulation of MITF expression, a mechanism that could contribute to its antitumor effects observed in vivo.

Our immunofluorescence analyses further revealed that both the total protein levels and phosphorylation status of key oncogenic signaling molecules—including ERBB2, BRAF, PI3K, AKT1, and mTOR—were significantly reduced in treated groups, with the most pronounced effects observed in the combination therapy. These pathways play critical roles in cell proliferation, survival, and tumor progression in melanoma [33]. The observed decrease in phosphorylation levels of BRAF, PI3K, AKT1, and mTOR is particularly relevant, as constitutive activation of these pathways has been strongly associated with melanoma growth and resistance to therapy [34]. By targeting multiple nodes within these signaling cascades, PTX and NCTD appear to exert a dual inhibitory effect on both upstream and downstream regulators, contributing to the observed reduction in tumor growth. These results are in line with previous reports demonstrating that simultaneous inhibition of the PI3K/AKT/mTOR and MAPK/ERK pathways can produce synergistic antitumor effects in melanoma models, offering a potential mechanistic explanation for the enhanced efficacy observed with combination therapy [35].

Transcriptomic analysis by RNA-seq provided additional insights into the molecular mechanisms underlying the observed antitumor effects. The number of DEGs was markedly higher in the intratumoral treatment groups, particularly in tumors treated with NCTD and the combination therapy, compared to intraperitoneal treatments. This finding underscores the greater transcriptomic impact of local drug delivery, likely due to higher intratumoral drug concentrations and more direct modulation of tumor-specific pathways [36]. Functional enrichment analyses using KEGG and Reactome databases revealed that the DEGs were significantly associated with pathways related to oxidative stress, immune response, protein translation, energy metabolism, and key oncogenic signaling cascades, including PI3K/AKT/mTOR, HIF-1, NF-κB, and MAPK pathways. Notably, intratumoral treatments showed enrichment in pathways associated with inflammatory response, stress adaptation, and extracellular matrix remodeling, suggesting that local therapy not only affects tumor cell-intrinsic pathways but may also modulate the tumor microenvironment [37,38]. Interestingly, hierarchical clustering analysis of all treatment groups, irrespective of administration route, revealed that samples tended to cluster according to the type of drug administered rather than the delivery method (Supplementary Figure S8). This suggests that the transcriptomic response in melanoma tumors was primarily driven by the pharmacological agent, highlighting the dominant effect of PTX, NCTD, or their combination on gene expression profiles.

To support the molecular and transcriptomic findings, in silico molecular docking analyses were performed to predict drug–target interactions between PTX, NCTD, and key proteins involved in melanoma progression. Both compounds exhibited favorable binding energies with several targets, including PI3K, AKT1, mTOR, BRAF, and ERBB2, all of which are critical regulators of cell proliferation and survival in melanoma [33,34,39,40]. The molecular docking analyses predicted that PTX and NCTD interact with catalytically and functionally important residues within the kinase domains or regulatory regions of these proteins. For example, PTX showed strong binding affinity for PI3K at residues His670 and Arg818, both located near the ATP binding site, while NCTD formed interactions with Gln1033 and Leu1036, suggesting inhibition of kinase activity. Similarly, both ligands exhibited interactions with residues within the activation loops of AKT1 and mTOR, supporting their capacity to interfere with signal transduction. The docking results complement the in vivo reductions observed in both total protein expression and phosphorylation levels, suggesting that direct molecular interactions may underpin the inhibition of PI3K/AKT/mTOR and MAPK/ERK pathways. Although experimental validation is warranted, these findings offer mechanistic insight into the antitumor effects observed in this melanoma model [41,42]. The molecular docking analysis predicted non-overlapping binding sites for pentoxifylline and norcantharidin on several key target proteins (Figure 11). Although these findings are based on computational models and should be interpreted with caution, such non-redundant interactions could potentially contribute to the observed drug synergy at the molecular level, by affecting distinct regulatory sites within shared signaling pathways.

To further contextualize the predicted interactions of PTX and NCTD, we benchmarked their docking scores against those of clinically validated or widely used inhibitors targeting the same proteins (Supplementary Table S2). While Vemurafenib (−10.5 kcal/mol), Rapamycin (−9.8 kcal/mol), Lapatinib (−8.3 kcal/mol), and MK-2206 (−7.7 kcal/mol) displayed the expected high-affinity binding to their respective targets (BRAF, mTOR, ERBB2, and AKT1), the docking scores of PTX and NCTD ranged from −6.01 to −7.44 kcal/mol (Table 1). This suggests that although their predicted binding affinities are lower than those of established inhibitors, they may still exert relevant interactions with these targets, especially in the context of multitarget modulation. These benchmarking comparisons support a cautious but plausible interpretation of PTX and NCTD as potential modulators of the PI3K/AKT/mTOR signaling pathway.

In addition, experimental data report an IC50 value of approximately 168 µM (1.68 × 10^−4^ M) for pentoxifylline’s inhibition of phosphodiesterase activity [43]. Using the thermodynamic relation ΔG = RT ln Ki at 298 K, this IC50 corresponds to a ΔG of about −5.14 kcal/mol. In comparison, the docking simulations performed in this study yielded binding energies for pentoxifylline ranging from −6 to −8 kcal/mol across different melanoma-related targets. While docking tends to overestimate binding affinities due to the static nature of the models and lack of dynamic cellular context, the predicted energies are within a plausible range relative to experimentally measured affinities. Similarly, norcantharidin (NCTD) has been shown experimentally to preferentially bind protein phosphatase 1 (PP1), disrupting the EZH2-PP1 interaction critical for epigenetic regulation [44] with NCTD demonstrating binding energies less than −5 kcal/mol for PP1. Also, a binding affinity range from −7.34 to −8.18 kcal/mol has been reported for the Mu-type opioid receptor and serine/threonine protein phosphatase 5, respectively [43]. These findings corroborate the docking predictions presented here, suggesting that both pentoxifylline and norcantharidin may engage relevant protein targets with biologically meaningful affinity. This benchmarking strengthens the confidence in our computational docking approach as a valuable tool for identifying potential molecular interactions underlying the antitumor effects of these compounds.

**Figure 12:**
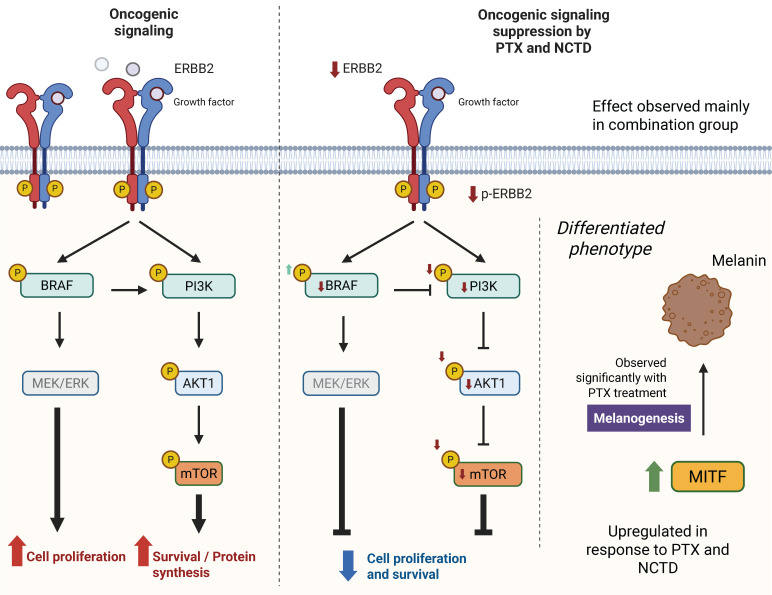
Schematic summary of the main signaling pathways modulated by pentoxifylline (PTX), norcantharidin (NCTD), and their combination in B16-F1 melanoma tumors. The diagram illustrates key proteins involved in the PI3K/AKT/mTOR and ERBB2/BRAF/MEK/ERK pathways and their downstream effects on cell proliferation, survival, and melanogenesis. Reduced phosphorylation (P) levels in treated groups are indicated, along with increased MITF expression and melanin production observed mainly in PTX-treated tumors. The most extensive suppression of oncogenic signaling was observed in the combination group.

Taken together, our data suggest that the antitumor effects of PTX and NCTD, both individually and in combination, are mediated through a multimodal mechanism involving inhibition of key oncogenic signaling pathways (PI3K/AKT/mTOR and BRAF/ERK), induction of tumor cell differentiation via MITF upregulation, and modulation of transcriptional programs linked to proliferation, metabolism, and stress response. The combination therapy consistently produced the most robust effects across all experimental approaches, including tumor growth inhibition, pathway suppression, and transcriptomic reprogramming. A schematic model summarizing the proposed molecular mechanisms and signaling alterations induced by the treatments is presented in Figure 12. This integrative figure highlights how PTX and NCTD may act synergistically to suppress oncogenic signaling, promote melanoma cell differentiation, and reduce tumor proliferation and survival. While further studies are needed to validate these findings in additional melanoma models and to explore the clinical translational potential [26,35,45], our results provide compelling preclinical evidence supporting the combined use of PTX and NCTD as a novel therapeutic strategy for melanoma management.

### Limitations

Although this study provides a comprehensive evaluation of the antitumor effects of pentoxifylline (PTX) and norcantharidin (NCTD) through in vivo, transcriptomic, and in silico approaches, several limitations should be acknowledged. Firstly, the in vivo experiments were conducted in a murine melanoma model (B16-F1), which, although widely used, may not fully recapitulate the complexity and heterogeneity of human melanoma. Differences in tumor microenvironment, immune responses, and genetic background between mice and humans may affect the translational applicability of the findings. Therefore, additional validation in human melanoma cell lines and patient-derived xenograft (PDX) models is necessary to strengthen the clinical relevance of the observed effects.

Secondly, the molecular docking analyses, while useful for predicting potential drug–target interactions, are inherently limited by their computational nature. Docking results provide theoretical binding affinities and interaction patterns but do not account for dynamic conformational changes, post-translational modifications, or cellular context that may influence actual binding in vivo. Experimental validation through biochemical assays such as surface plasmon resonance, kinase inhibition assays, or co-immunoprecipitation is required to confirm the predicted interactions and assess functional consequences.

Lastly, this study did not evaluate pharmacokinetic parameters or drug distribution of the PTX–NCTD combination, which are critical for the development of effective and safe therapeutic strategies. Future studies should integrate pharmacological and toxicological evaluations to better inform clinical translation.

## 4. Methods and Materials

### 4.1. Cell Culture

A vial of cryopreserved melanoma B16-F1 cells (ATCC^®^ CRL-6323™) from the American Type Culture Collection (ATCC^®^, Manassas, VA, USA) was thawed in a 37 °C water bath for 30 s and immediately diluted in Dulbecco’s Modified Eagle Medium ME-019 (DMEM; In Vitro, S.A., Mexico City, Mexico) supplemented with with 10% Fetal Bovine Serum (FBS; Gibco^®^, Life Technologies™, Carlsbad, CA, USA). The cell suspension was centrifuged at 100 RCF for 3 min, after which the supernatant was removed, and the cell pellet was resuspended in DMEM supplemented with 10% FBS. The cells were then transferred to 75 cm^2^ cell culture-treated flasks with filter caps (Thermo Scientific™, Thermo Fisher Scientific, Waltham, MA, USA) containing DMEM supplemented with 10% FBS. The cells were cultured at 37 °C in a humidified incubator with 5% CO_2_ and 95% relative humidity. After 24 h, cell adhesion to the flask was confirmed, and the culture medium was replaced to remove any remaining cryopreservation medium and non-viable cells. Once the cells reached greater than 80% confluency, they were treated with Trypsin-EDTA (Gibco^®^, Life Technologies™, Carlsbad, CA, USA) solution and then recovered for subsequent experimental procedures.

### 4.2. Establishment of the Murine Melanoma Model

Inbred DBA/2J mice (6–8 weeks old) were obtained from the Environmental Toxicology Laboratory of the Escuela Nacional de Ciencias Biológicas (ENCB), Instituto Politécnico Nacional. The animals were housed in polycarbonate cages with sawdust bedding under controlled conditions (23 °C, 40–60% humidity) and maintained with a 12 h light–12 h dark cycle. Food and water were provided ad libitum, with rodents receiving NUTRICUBOS^®^ Rodent Chow (Cargill^®^, Tehuacán, Puebla, Mexico; SAGARPA Authorization A-0207-246) and potable water. The specimens were managed according to Article 38 and Chapter V of Directive 2010/63/EU of the European Parliament and of the Council of 22 September 2010 on the protection of animals used for scientific purposes ([46], accessed on June 2021). The current study was reviewed and approved by the ENCB Bioethics Committee, with the license number CEI-ENCB-ZOO-021-2020.

This study included two main experimental groups: (1) an intraperitoneal (IP) treatment group, in which 7 × 10^5^ cells were subcutaneously implanted on the right flank of each mouse, and therapeutic agents were administered intraperitoneally; (2) an intratumoral (IT) treatment group, in which 7 × 10^5^ cells were subcutaneously implanted on the right flank of each mouse, and drugs were administered directly into the tumor. On Day 0, cells were implanted, and once a visible and palpable tumor developed (Supplementary Figure S1), treatment groups were sex-matched and randomly assigned, and pharmacological treatment was initiated.

### 4.3. Pharmacological Treatments

Norcantaridin (NCTD, CAS No. 5442-12-6; Sigma^®^, Merck KGaA, St. Louis, MO, USA) and pentoxifylline (PTX, CAS No. 6493-05-6; Sigma^®^, Merck KGaA, St. Louis, MO, USA) were dissolved in either sterile saline solution or sterile dimethyl sulfoxide (DMSO), depending on the route of administration—intraperitoneal (IP) or intratumoral (IT), respectively. For the IP treatment group, animals were randomly assigned to the following subgroups: Subgroup 1 (Control), receiving sterile saline solution; Subgroup 2, receiving PTX at 60 mg/kg; Subgroup 3, PTX at 30 mg/kg; Subgroup 4, NCTD at 3 mg/kg; Subgroup 5, a combination of PTX at 60 mg/kg and NCTD at 3 mg/kg; Subgroup 6, PTX at 60 mg/kg combined with NCTD at 0.75 mg/kg; and Subgroup 7, NCTD at 0.75 mg/kg. For the IT treatment group, animals were divided into four subgroups as follows: Subgroup 1 (Control), receiving DMSO; Subgroup 2, PTX at 60 mg/kg; Subgroup 3, NCTD at 3 mg/kg; and Subgroup 4, PTX at 60 mg/kg combined with NCTD at 3 mg/kg.

The doses were administered over a period of 8 days, as depicted in Figure 1a, and determined based on previous studies from our research group [11,47], ensuring the absence of systemic toxic effects.

Mice were weighed using a digital scale, and tumor growth was monitored with a digital caliper from the start to the end of the treatment period. Tumor volume was calculated using the following formula:

V=(lenght × width2)2

where *length* and *width* are the longest and shortest dimensions of the tumor, respectively [48]. To assess treatment efficacy over time, relative tumor volume (*RTV*) was calculated for each individual mouse as the ratio between the tumor volume at a given time point (*V_x_*) and the tumor volume at the beginning of treatment (*V*_0_, D6):

RTV=VxV0

This normalization allowed for the comparison of tumor growth dynamics across individuals and treatment groups, regardless of initial tumor size.

Portions of tumor tissue designated for nucleic acid extraction were immediately frozen at −80 °C until further processing, while the remaining tumor samples were collected and fixed in buffered formalin for subsequent immunofluorescence analysis.

### 4.4. Total RNA Extraction and cDNA Library Preparation

Total RNA was extracted from tumor samples using the PureLink^®^ RNA Mini Kit (Thermo Fisher Scientific, Waltham, MA, USA), following the manufacturer’s protocol. The extracted RNA from tumor samples was subsequently used for cDNA library preparation. cDNA libraries from tumors of mice were prepared using the Collibri™ 3′ mRNA Library Prep Kit for Illumina™ (Thermo Fisher Scientific, Waltham, MA, USA), following the recommended guidelines for RNA purification, amplification, and library construction for bulk RNA-Seq analysis. The cDNA libraries prepared in the laboratory were stored in an ultrafreezer at −80 °C.

### 4.5. RNA Sequencing and Data Acquisition

The cDNA libraries were submitted to Novogene Co., Ltd. (Sacramento, CA, USA) for RNA sequencing. Sequencing was carried out on the Illumina^®^ HiSeq PE150 platform (Illumina, San Diego, CA, USA), generating paired-end reads of 150 base pairs in length. The sequencing depth was selected to ensure comprehensive coverage of the transcriptome. Raw sequencing data were delivered in FASTQ format and subjected to quality control analysis before being processed for downstream bioinformatics.

### 4.6. RNA-Seq Data Analysis Pipeline

The raw RNA-Seq data were processed and analyzed using a comprehensive bioinformatics pipeline. First, the raw FASTQ files were assessed for quality using FastQC [49] (version 0.12.1 released). Low-quality reads and adapter sequences were removed using Trimmomatic [50] (version 0.39). Cleaned reads were then aligned to the Ensembl release reference mouse genome (version GRCm39) using the STAR aligner [51] (version 2.7.11b). The resulting BAM files were sorted and indexed, and gene expression levels were quantified using STAR (version 2.7.11b). Differential expression analysis was performed using DESeq2 [52] (version 1.40.2), with statistical significance determined at a false discovery rate (FDR) of <0.05. Gene ontology (GO) and pathway enrichment analyses were conducted using clusterProfiler [53] (version 4.15.1) to interpret biological functions associated with differentially expressed genes. All analyses were performed in R [54] (version 4.4.1) and using Bioconductor packages (KEGG and Reactome analyses) in R Studio [55] (Version 2025.05.1+513).

### 4.7. Tumor Tissue Immunofluorescence

Tumor tissue samples were embedded in paraffin and sectioned at 3–4 µm thickness. The slides were air-dried for at least 12 h at 37 °C. The sections were deparaffinized using xylene and subsequently hydrated through sequential alcohol and water baths. Antigen retrieval was performed by heating the sections at 95 °C in citrate buffer for at least 40 min. The samples were then allowed to cool for 20 min at room temperature, followed by a wash in PBS buffer. Fluorochrome-labeled specific antibodies for antigen detection, including ERBB2, AKT-1, mTOR, BRAF, PI3K (both phosphorylated [active] and non-phosphorylated [inactive] forms), and MITF, were used at a 1:3000 dilution. After antibody staining, the sections were incubated with DAPI for nuclear counterstaining. Finally, the samples were mounted with glycerol and analyzed using a confocal laser scanning microscope (LSM 5 EXCITER, Carl Zeiss^®^, Oberkochen, Baden-Württemberg, Germany). Image analysis (mean fluorescence intensity) was performed using ImageJ [56] (Version 1.54g).

### 4.8. Molecular Docking Studies

To elucidate the interactions of PTX and NCTD with key proteins involved in melanoma progression, oncogenic signaling, angiogenesis, and the tumor microenvironment, molecular docking studies were conducted. The structures of PTX and NCTD were obtained from the PubChem database [57]. The crystal structures of the proteins analyzed were retrieved from the Protein Data Bank (PDB) [58]. Protein–ligand masked docking studies were performed using Autodock Vina software (Version 1.1.2), as previously reported [42]. The top-ranked complex from each study was analyzed to identify molecular interactions, following established protocols [42]. Visualization was performed using MAESTRO (Version 13.1.141) and PyMOL software (Version 2.5.3), as previously described [42,59].

### 4.9. Statical Analysis

The results were analyzed by ANOVA, followed by the post hoc Dunnett test. All *p*-values ≤ 0.05 were considered statistically significant. R (version 4.4.1) and R Studio software (Version 2025.05.1+513) were used for statistical analysis and graphic representation.

## 5. Conclusions

This study provides comprehensive preclinical evidence demonstrating that pentoxifylline (PTX) and norcantharidin (NCTD), administered either individually or in combination, effectively suppressed melanoma progression in a murine model using B16-F1 cells implanted in DBA/2J mice. Both intraperitoneal and intratumoral administration routes were evaluated, with local intratumoral delivery showing superior efficacy in reducing tumor volume and inducing broader transcriptomic changes. At the molecular level, treatment with PTX and NCTD resulted in the downregulation of key oncogenic pathways, including the PI3K/AKT/mTOR and BRAF/ERK pathways, accompanied by a reduction in both total and phosphorylated protein levels of primary signaling mediators. Additionally, PTX promoted tumor cell differentiation, as evidenced by increased MITF expression and enhanced melanin production, suggesting a potential shift towards a less aggressive melanoma phenotype. RNA-seq analysis further revealed that the treatments modulated genes involved in cell proliferation, metabolism, immune response, and stress adaptation, with functional enrichment pointing to significant alterations in cancer-related, metabolic, and inflammatory pathways. Finally, in silico molecular docking studies supported these findings by predicting binding interactions of PTX and NCTD with multiple proteins involved in melanoma growth and survival, providing a plausible mechanistic basis for the observed in vivo effects. Together, these findings highlight the therapeutic potential of PTX and NCTD, particularly when combined, as promising candidates for melanoma treatment strategies aimed at simultaneously modulating multiple oncogenic pathways. Further studies, including in vitro mechanistic validation and clinical translation approaches, are warranted to explore their potential role in future melanoma therapy.

## Figures and Tables

**Table 1:** Predicted binding energies of PTX and NCTD (docking scores) with key oncogenic proteins in human and murine models.

Protein	PDB ID	Ligand	Binding Energy (ΔG, kcal/mol)	Binding Site/Residues
Epithelial–Mesenchymal Transition (EMT)				
CDH1	3Q2V	NCTD	−4.76	Tyr175, Asp168. Leu167
		PTX	−4.96	Met193, Glu190, His180
LIM domain-binding protein 1	6PTL	NCTD	−5.47	Gln125, Gly144, Glu143
		PTX	−5.23	Ile165, Phe72, Ile70, Met128, Tyr91, Ile95, Ser94, His132
FZD8	1IJY	NCTD	−6.16	Gly113, Val114, Cys115, Val109, Pro108, Lys77
		PTX	−6.44	Ser78, Cys115, Gly113, Lys77, Pro108, Lys74, Val109
TGF-β1	1KLC	NCTD	−4.79	Arg25, Lys37, Phe24, His34
		PTX	−5.00	Arg25, Phe24, His34
CTNND1	3L6X	NCTD	−5.52	Asp510, Arg383
		PTX	−5.20	Lue620, Glu521, Arg326, Arg329, Val514, Asn517
LFA-1	3F74	NCTD	−4.91	Gly262, Leu289, Ile288, Asp290
		PTX	−5.06	Thr164, Tyr166, Ser165, Thr231, Gly128
CXCL12γ	6EHZ	NCTD	−5.11	Arg12, Cys50, Val49
		PTX	−5.06	Arg12, Cys50, Arg47, Val39, Gln46
Claudin-2	4YYX	NCTD	−6.18	Ile38, Ile36, Gly35, Phe34, Leu95, Arg96
		PTX	−6.47	Asn72, Arg74, Glu51, His46, Gly40, Val55
Cell Proliferation				
PIK3CA	4A55	NCTD	−5.72	Gln1033, Leu1036, Glu1037, Thr1040
		PTX	−6.50	His670, Arg662, Asn170, Arg818, Cys838, Leu839
AKT1	3O96	NCTD	−5.99	Leu360, Tyr340, Arg367, Arg346, Leu347, Pro358, Phe349, Tyr350
		PTX	−6.10	Leu52, Pro51, Asp325, Gly327
mTOR	6BCX	NCTD	−6.60	His1398, Trp2313, Trp2304, Ala2386
		PTX	−6.88	Gln1901, His2410, Asp2412
ERK2	6GJD	NCTD	−6.60	Val39, Lys54, Thr105
		PTX	−6.70	Leu156, Lys54, Ala52, Ile31, Ile86
Melanoma Stem Cell Marker				
CD117	1PKG	NCTD	−6.62	Ala621, Cys809, Leu799, Lys623, Thr670, Val603
		PTX	−7.40	Ala597, Asp810, Asp677, Cys809, Gly596, Leu799, Lys623, Thr6670, Val603
CD117 (extracellular domain)	2EC8	NCTD	−4.90	Gln346, Pro343
		PTX	−5.24	Glu228, Glu368, Leu222, Leu223, Lys342, Thr230, Thr342
KITLG	1EXZ	NCTD	−5.56	Ala147, Arg13, Gly151, Tyr150, Val73, Val170
		PTX	−6.01	Ala147, Arg13, Arg14, Ile152, Ser11, Thr71
KDM5B	5A3P	NCTD	−6.52	Leu716, Met701, Phe700
		PTX	−5.57	Asp77, Leu81, Phe438, Pro439, Val440
Key Drivers of Melanocytic Transformation				
MITF	4ATK	NCTD	−4.66	Asp252
		PTX	−5.53	Ala249, Lys233, Met239, Pro232, Tyr253
B-RAF	4MNF	NCTD	−6.22	Cys532, Phe583, Trp531
		PTX	−7.44	Lys483, Ala481, Asp594, Gly466, Lau514, Phe583, Thr529
RAF-MEK1 complex	4MNE	NCTD	−6.34	Leu74, Leu197, Met146
		PTX	−7.33	Asp208, Gly210, Lys97, Met219, Leu215, Phe209
ERBB2	2A91	NCTD	−6.01	Pro279, Phe270, Ans467
		PTX	−6.73	Asn467, Gly443, Leu28, Tyr280, Val4
NRAS GTPase	6E6H	NCTD	−5.91	Asp13, Gly15, Lys16, Ser17, Val14
		PTX	−6.61	Asp13, Ala18, Ala146, Lys117, Lys147, Phe28, Ser17
Vascularization and Angiogenesis				
HIF1A	3HQR	NCTD	−7.83	Arg383, Ile327, His374, Thr387, Val376
		PTX	−6.34	Arg322, Ile251, Leu240, Tyr310, Val241, Val314
VEGF-A	2VPF	NCTD	−4.53	Ile46, Phe36, Phe47
		PTX	−4.74	Asp63, Cys61, Cys68, Glu64, Lys107
EGF	1IVO	NCTD	−3.51	Lys48
		PTX	−4.43	Gly36, Trp49, Trp50
TWIST1	2MVJ	NCTD	−4.01	Ala9
		PTX	−4.04	Ala6, Ala9, Gly89, Lys10, Ser11
PDPK1	1H1W	NCTD	−6.51	Phe93, ser94, Val127
		PTX	−6.41	Glu130, Lys111, Lys123, Leu113, Ser94
PTGS2	1PXX	NCTD	−6.13	Val291
		PTX	−7.33	Arg44, Leu152, Lys468
Matrix metalloproteinase-1	966C	NCTD	−5.90	Thr241, Val246
		PTX	−5.53	Arg214, Asn205, Asn211, his132, his213, Lys136
Matrix metalloproteinase-2	1QIB	NCTD	−6.53	Leu218, Thr227, Tyr223
		PTX	−6.83	Leu164, Tyr223, Val198
Matrix metalloproteinase-9	5UE3	NCTD	−6.54	Arg249, His228, Leu222, Leu243, Tyr248
		PTX	−7.02	Arg249, His257, Leu243, Thr251
NF-κB (p50 subunit)	1SVC	NCTD	−5.42	Lys147
		PTX	−5.34	Lys147, Phe151, Thr205, Val150

## Data Availability

The raw and proceeded data from RNA-seq supporting the conclusions of this article are available in the GEO (Gene Expression Omnibus) NCBI Repository: GSE301760. The raw data supporting the conclusions of this article will be made available by the authors on request.

## Supplementary Materials

The following supporting information can be downloaded at https://www.mdpi.com/article/10.3390/ijms26157522/s1.

## Abbreviations

The following abbreviations are used in this manuscript:

CDH1	E-cadherin
FZD8	Frizzled-8 receptor
TGF-β1	Transforming growth factor beta 1
CTNND1	p120-catenin
LFA-1	CD11a/CD18 integrin complex = ITGAL/ITGB2
CXCL12γ	C-X-C motif chemokine ligand 12, gamma subunit
Claudin-2	CLDN2
PIK3CA	PI3K catalytic subunit p110α
AKT1	AKT serine/threonine protein kinase 1
mTOR	Mechanistic target of rapamycin
ERK2	MAPK1 protein
CD117	KIT receptor
KITLG	Stem Cell Factor (KIT ligand)
KDM5B	Histone demethylase
MITF	Microphthalmia-associated TF
B-RAF	B-Raf proto-oncogene serine/threonine-protein kinase
ERBB2	HER2 receptor
HIF1A	Hypoxia-inducible factor 1-alpha
VEGF-A	Vascular endothelial growth factor A
EGF	Epidermal growth factor
TWIST1	Twist-related protein 1
PTGS2	Cyclooxygenase-2
PDPK1	3-phosphoinositide-dependent protein kinase-1
NF-κB	NF-κB complex
GO	Gene Ontology
KEGG	Kyoto Encyclopedia of Genes and Genomes
Reactome	Reactome Pathway Database
GSEA	Gene Set Enrichment Analysis
SD	Standard deviation
D	Day (D6, Day 6)
ΔG	Binding free energy
