# Supplementary material for: Pentoxifylline and Norcantharidin Synergistically Suppress Melanoma Growth in Mice: A Multi-Modal In Vivo and In Silico Study
Source: Int J Mol Sci. 2025 Aug 4;26(15):7522. doi: 10.3390/ijms26157522 (PMC12347239; doi:10.3390/ijms26157522)
Supplement: Supplementary file 1 [file ijms-26-07522-s001.zip › Figure_S1.pdf]

MICE: DBA/2J, 6-8 weeks old

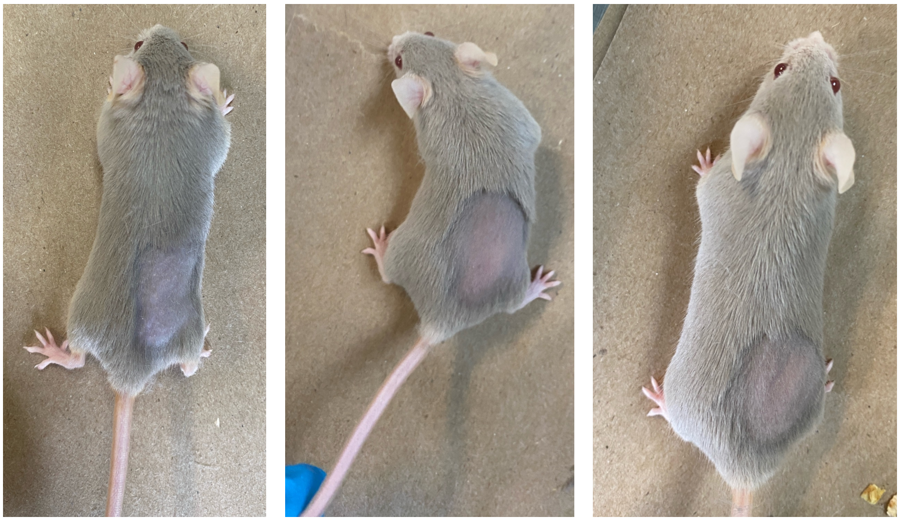

D0

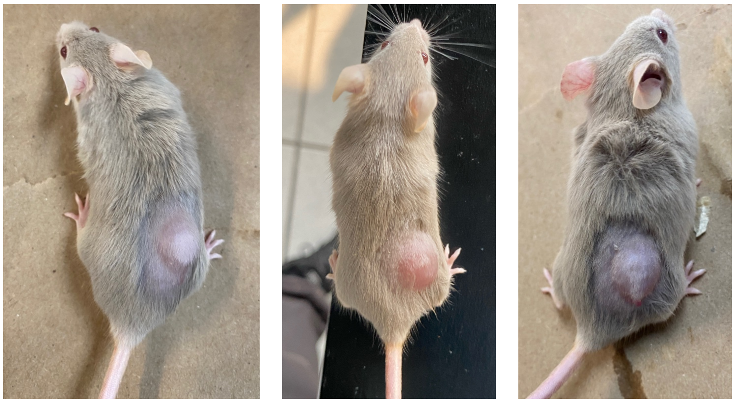

D0: Subcutaneous injection of B16-F1 melanoma cells

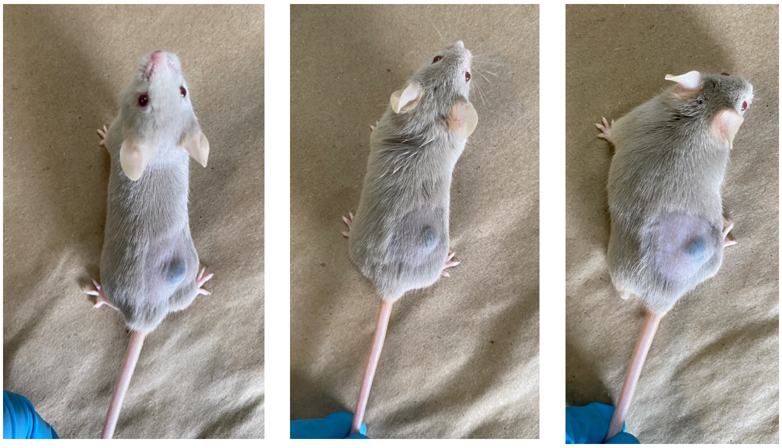

D6: treatment initiation

Figure S1. Representative DBA/2J mice at baseline (D0) and day 6 (D6) showing tumor development prior to treatment initiation. Representative DBA/2J mice on day 0 (D0) after shaving, followed by subcutaneous injection/implantation of B16-F1 melanoma cells into the right flank. Lower panels show the same mice on day 6 (D6), with visible tumor development reaching approximately 50 mm^3 in volume, marking the starting point for treatment administration.
