# Supplementary material for: Pentoxifylline and Norcantharidin Synergistically Suppress Melanoma Growth in Mice: A Multi-Modal In Vivo and In Silico Study
Source: Int J Mol Sci. 2025 Aug 4;26(15):7522. doi: 10.3390/ijms26157522 (PMC12347239; doi:10.3390/ijms26157522)
Supplement: Supplementary file 1 [file ijms-26-07522-s001.zip › Figure_S2.pdf]

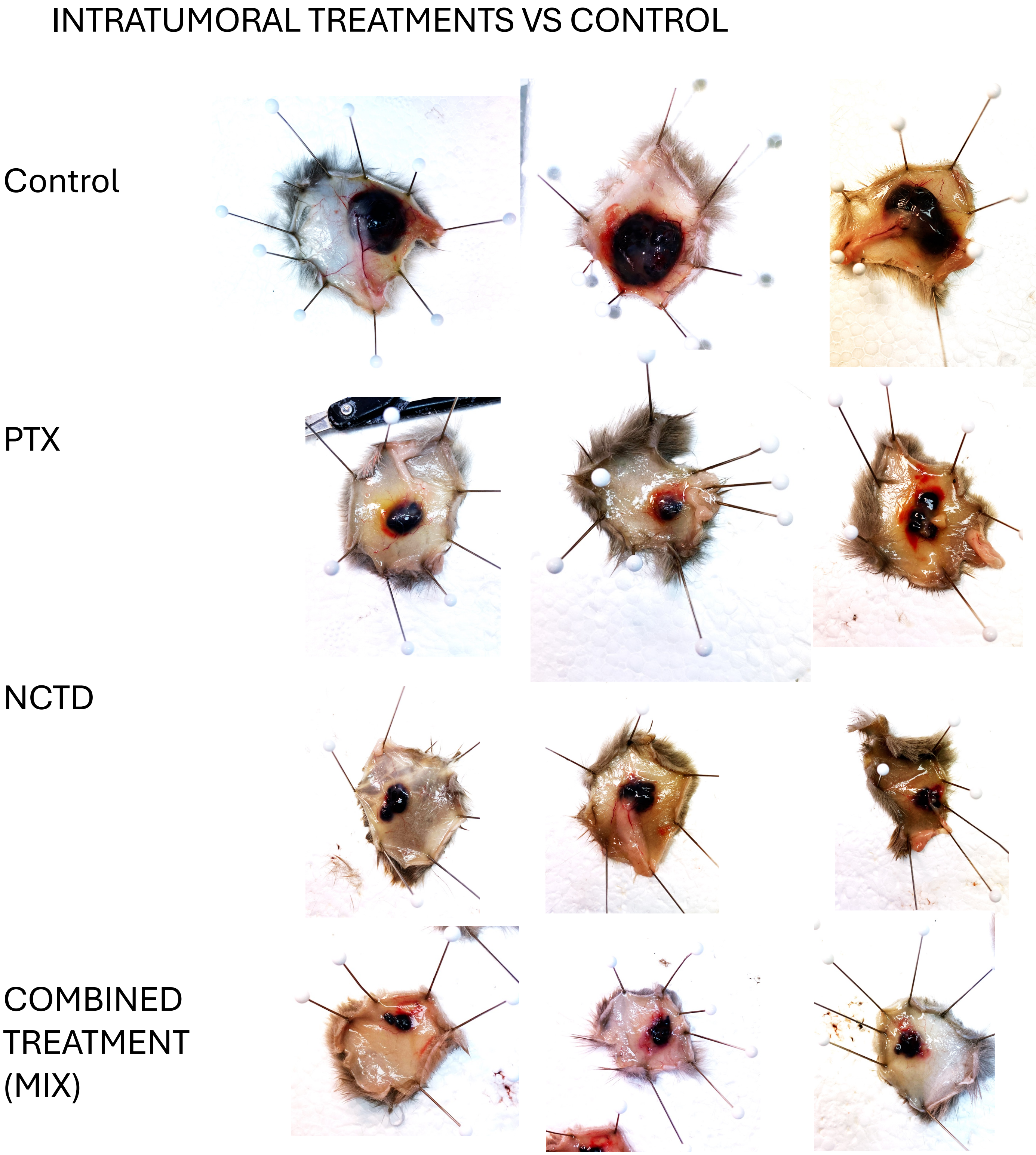

Figure S2. Representative images of excised tumors from intratumorally treated mice after euthanasia, showing tumor size differences across treatment groups. Representative images of excised tumors from melanoma-bearing mice following euthanasia, showing tumor size differences across the intratumoral treatment groups. From top to bottom: Control, PTX (60 mg/kg), NCTD (3 mg/kg), and the combination treatment (PTX + NCTD).
