# Supplementary material for: Pentoxifylline and Norcantharidin Synergistically Suppress Melanoma Growth in Mice: A Multi-Modal In Vivo and In Silico Study
Source: Int J Mol Sci. 2025 Aug 4;26(15):7522. doi: 10.3390/ijms26157522 (PMC12347239; doi:10.3390/ijms26157522)
Supplement: Supplementary file 1 [file ijms-26-07522-s001.zip › Figure_S5.pdf]

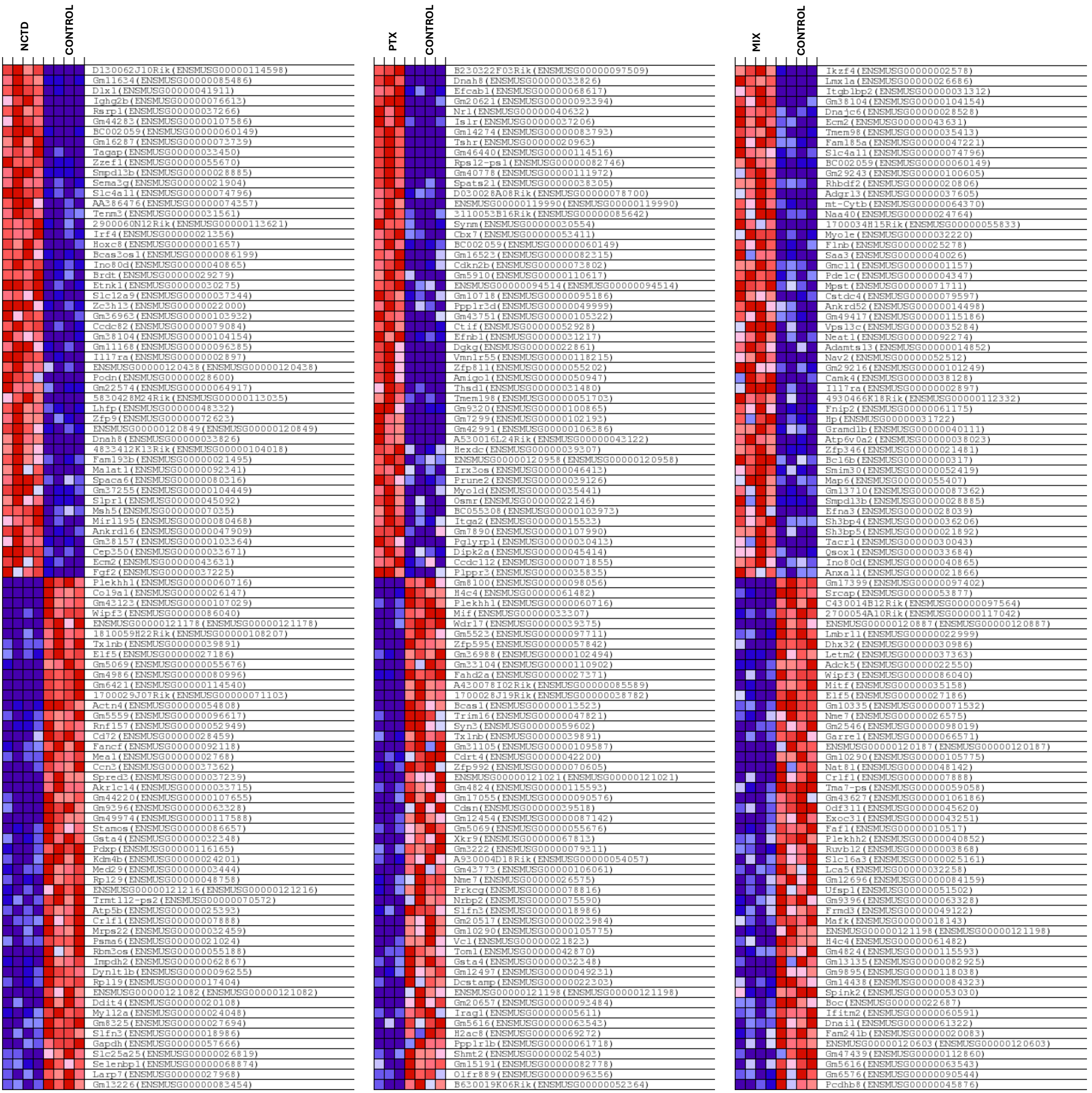

FIGURE S5. Hierarchical clustering and heatmap visualization of differentially expressed genes (DEGs) in intratumoral treatment groups. Hierarchical clustering and heatmap visualization of differentially expressed genes (DEGs) in intratumoral treatment groups. Heatmaps from GSEA-GO enrichment analyses display the top 50 features for each phenotype based on read count data. Gene expression profiles are shown for NCTD_IT vs CONTROL_IT, PTX_IT vs CONTROL_IT, and MIX_IT vs CONTROL_IT. Each row represents a single gene, and each column corresponds to a tumor sample (n = 3-4 per group). The color scale indicates relative gene expression levels, with red representing upregulation and blue indicating downregulation.
