# Supplementary material for: Pentoxifylline and Norcantharidin Synergistically Suppress Melanoma Growth in Mice: A Multi-Modal In Vivo and In Silico Study
Source: Int J Mol Sci. 2025 Aug 4;26(15):7522. doi: 10.3390/ijms26157522 (PMC12347239; doi:10.3390/ijms26157522)
Supplement: Supplementary file 1 [file ijms-26-07522-s001.zip › Figure_S7.pdf]

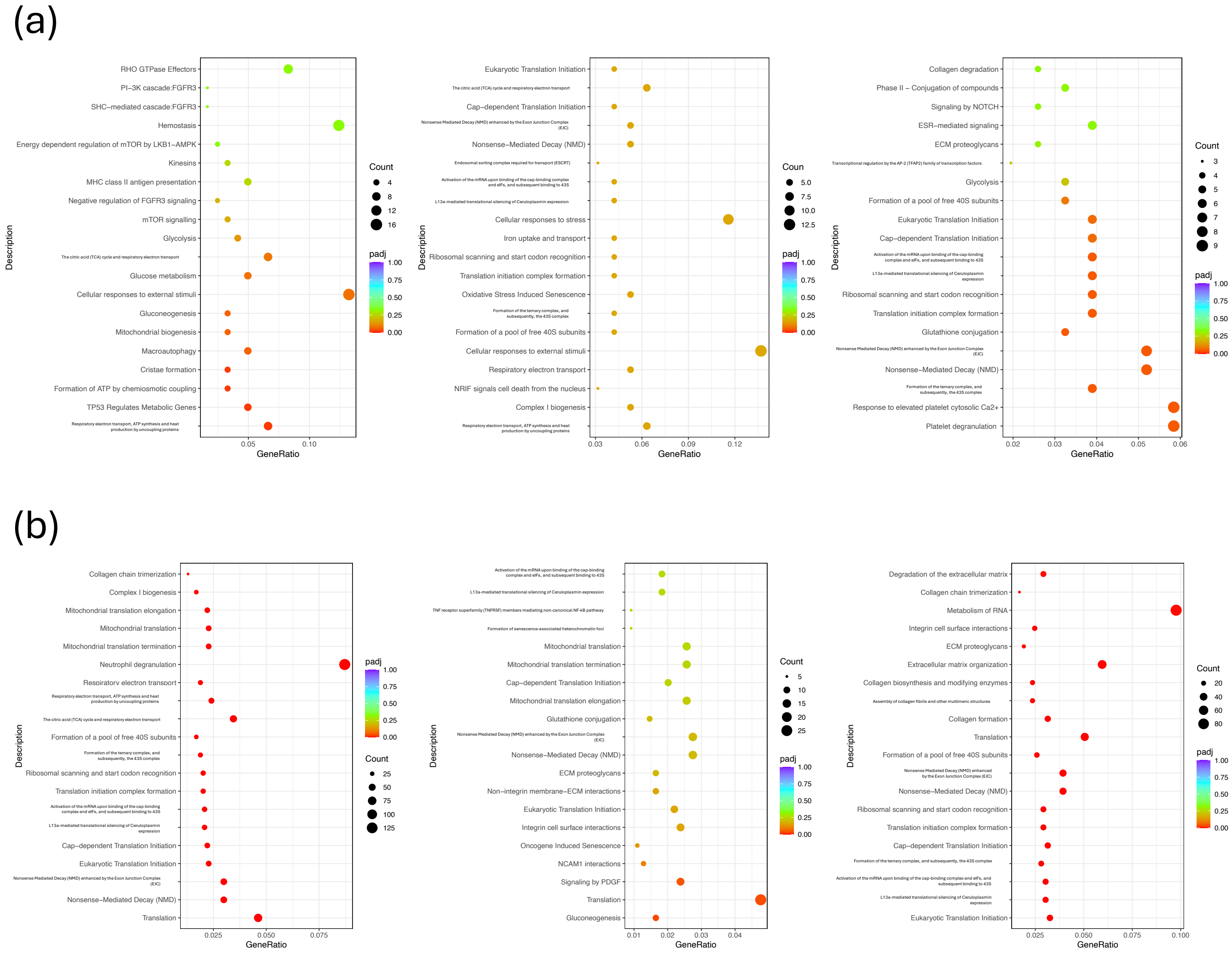

Figure S7. Reactome pathway enrichment analysis of differentially expressed genes (DEGs) induced by treatments. (a) Enriched pathways for intraperitoneal administration groups (NCTD_IP, PTX_IP, and MIX_IP vs CONTROL_IP), left to right respectively. (b) Enriched pathways for intratumoral administration groups (NCTD_IT, PTX_IT, and MIX_IT vs CONTROL_IT), left to right respectively. Dot plots display the most significantly enriched Reactome pathways, with dot size indicating the number of DEGs associated with each pathway and color representing the adjusted *p*-value (padj). Pathways are ranked by statistical significance.
