# Supplementary material for: Pentoxifylline and Norcantharidin Synergistically Suppress Melanoma Growth in Mice: A Multi-Modal In Vivo and In Silico Study
Source: Int J Mol Sci. 2025 Aug 4;26(15):7522. doi: 10.3390/ijms26157522 (PMC12347239; doi:10.3390/ijms26157522)
Supplement: Supplementary file 1 [file ijms-26-07522-s001.zip › Figure_S8.pdf]

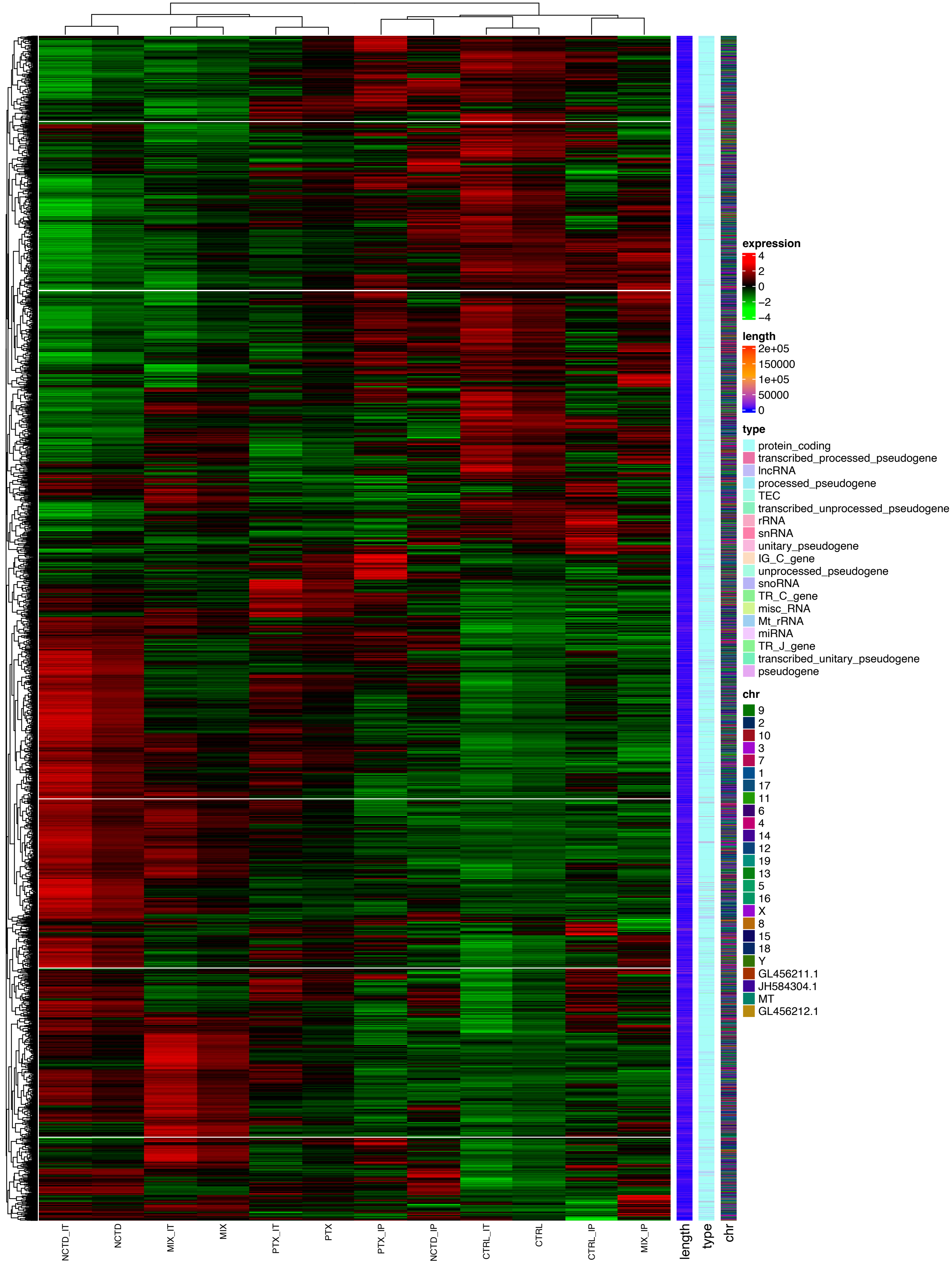


**FIGURE S8. Hierarchical clustering and heatmap of DEGs across all treatment groups and conditions.** Hierarchical clustering and heatmap visualization of differentially expressed genes (DEGs) across all treatment groups. The panel shows the overall clustering of all samples (intraperitoneal and intratumoral groups combined). Also it is display clustering by treatment type: Control, PTX (both doses and routes), NCTD (both doses and routes), and the combination treatment (PTX + NCTD), highlighting global gene expression patterns associated with each drug condition.
