# Supplementary material for: Pentoxifylline and Norcantharidin Synergistically Suppress Melanoma Growth in Mice: A Multi-Modal In Vivo and In Silico Study
Source: Int J Mol Sci. 2025 Aug 4;26(15):7522. doi: 10.3390/ijms26157522 (PMC12347239; doi:10.3390/ijms26157522)
Supplement: Supplementary file 1 [file ijms-26-07522-s001.zip › Table S1.pdf]

Table S1. Post hoc Tukey HSD pairwise comparisons for tumor volume between treatments and administration routes (IP vs IT) at day 9.

Group 1	Group 2	Mean Difference	Adjusted p-value	95% CI Lower	95% CI Upper	Significant (Yes/No)	Significance Level
CONTROL (IP)	CONTROL (IT)	0.5905	0.9773	-2.1964	3.3774	False	ns
CONTROL (IP)	NCTD 3 (IP)	-6.3157	0.0	-9.1027	-3.5288	True	***
CONTROL (IP)	PTX 60 (IP)	-7.2763	0.0	-10.0633	-4.4894	True	***
CONTROL (IP)	PTX 60 + NCTD 3 (IP)	-8.1587	0.0	-10.9456	-5.3718	True	***
CONTROL (IT)	NCTD 3 (IP)	-6.9062	0.0	-9.6932	-4.1193	True	***
CONTROL (IT)	PTX 60 (IP)	-7.8668	0.0	-10.6538	-5.0799	True	***
CONTROL (IT)	PTX 60 + NCTD 3 (IP)	-8.7492	0.0	-11.5362	-5.9623	True	***
NCTD 3 (IP)	PTX 60 (IP)	-0.9606	0.8767	-3.7475	1.8263	False	ns
NCTD 3 (IP)	PTX 60 + NCTD 3 (IP)	-1.843	0.3638	-4.6299	0.9439	False	ns
PTX 60 (IP)	PTX 60 + NCTD 3 (IP)	-0.8824	0.9066	-3.6693	1.9046	False	ns

Two-way ANOVA analyses. Significance levels: $p < 0.001$ (***). Adjusted p-values were used after correction for multiple comparisons. ns, not significance.
