# Supplementary material for: Pentoxifylline and Norcantharidin Synergistically Suppress Melanoma Growth in Mice: A Multi-Modal In Vivo and In Silico Study
Source: Int J Mol Sci. 2025 Aug 4;26(15):7522. doi: 10.3390/ijms26157522 (PMC12347239; doi:10.3390/ijms26157522)
Supplement: Supplementary file 1 [file ijms-26-07522-s001.zip › Table_S2.pdf]

Table S2. Reference compounds (positive controls).

<table>
<tr><th>Protein</th><th>Validated ligand / inhibitor</th><th>Clinical or experimental use</th><th>Binding energy: Kcal/mol</th></tr>
<tr><td>BRAF</td><td>Vemurafenib (PLX4032) - Pubchem ID: 42611257
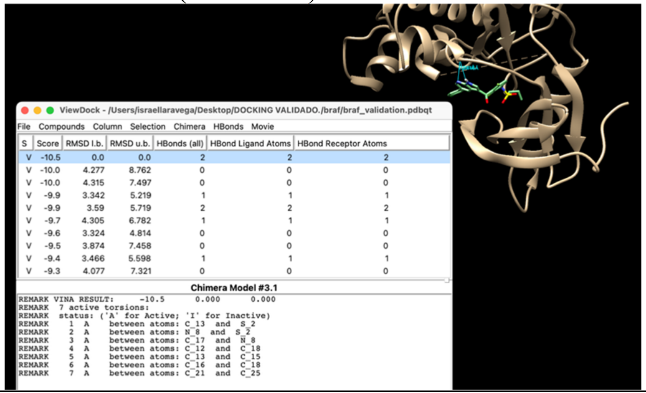
</td><td>Selective BRAF V600E inhibitor, approved for melanoma</td><td>-10.5</td></tr>
<tr><td>AKT1</td><td>MK-2206 - Pubchem ID: 24964624
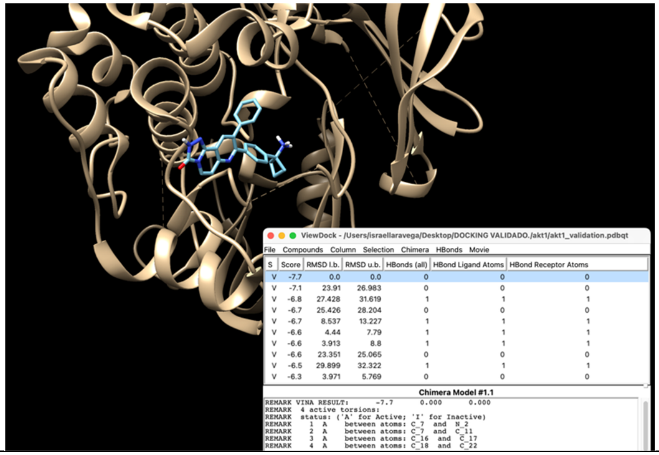
</td><td>Allosteric AKT inhibitor, widely used in preclinical studies</td><td>-7.7</td></tr>
</table>

<table>
<tr><td>mTOR</td><td>Rapamicina (Sirolimus) - Pubchem ID: 5284616
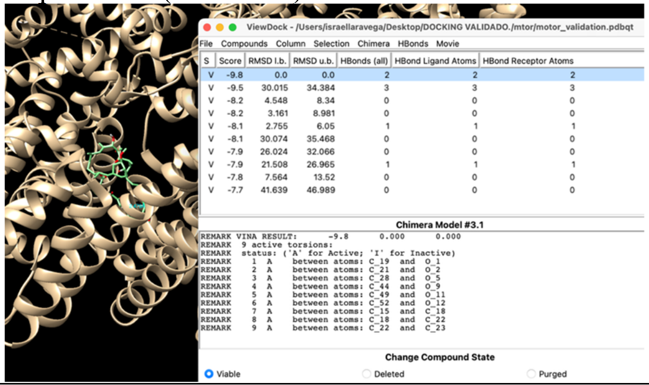
</td><td>Classic mTORC1 inhibitor, widely validated.</td><td>-9.8</td></tr>
<tr><td>ERBB2</td><td>Lapatinib - PubChem ID: 208908
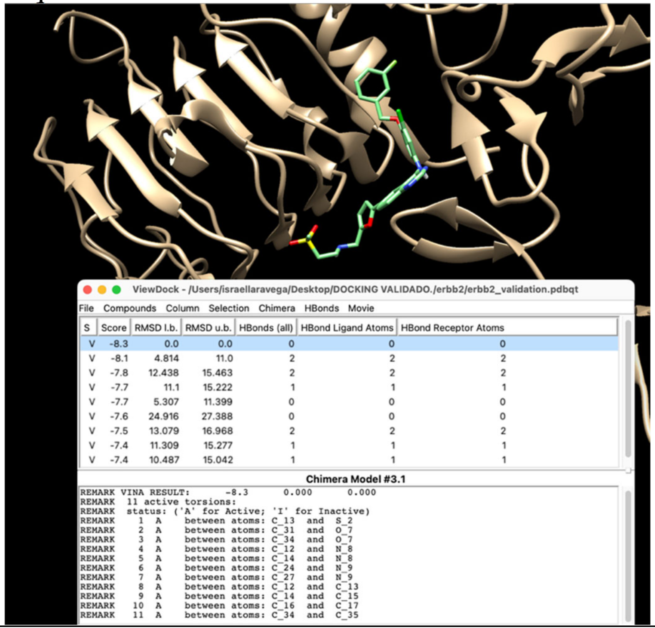
</td><td>Dual EGFR and HER2 (ERBB2) inhibitor, approved for HER2-positive breast cancer</td><td>-8.3</td></tr>
</table>
