# Supplementary figures and images for: Pentoxifylline and Norcantharidin Synergistically Suppress Melanoma Growth in Mice: A Multi-Modal In Vivo and In Silico Study
Source: Int J Mol Sci. 2025 Aug 4;26(15):7522. doi: 10.3390/ijms26157522 (PMC12347239; doi:10.3390/ijms26157522)

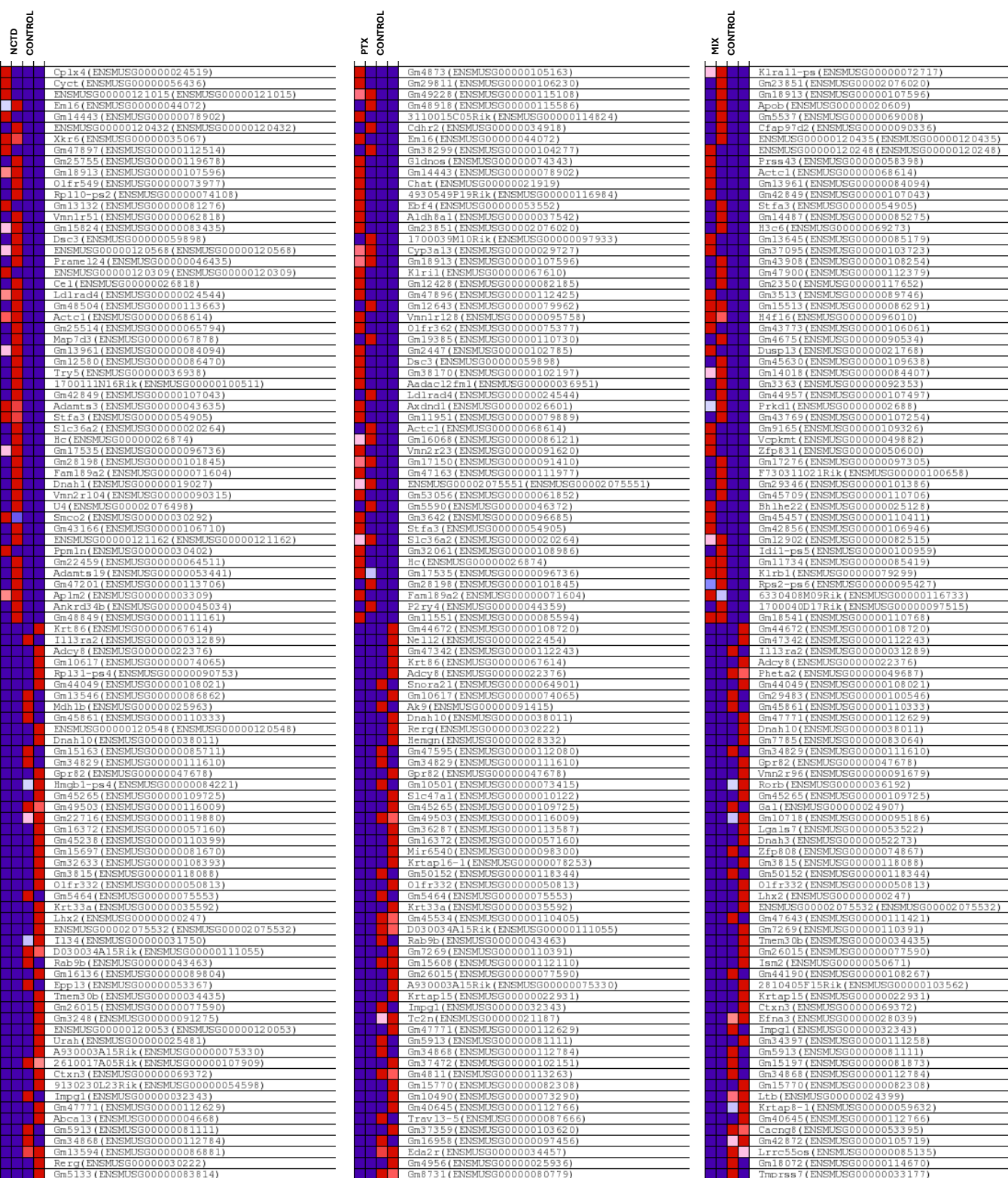

Supplement: Supplementary file 1 [file ijms-26-07522-s001.zip › Figure_S4.pdf]
