# Supplementary material for: Pentoxifylline and Norcantharidin Synergistically Suppress Melanoma Growth in Mice: A Multi-Modal In Vivo and In Silico Study
Source: Int J Mol Sci. 2025 Aug 4;26(15):7522. doi: 10.3390/ijms26157522 (PMC12347239; doi:10.3390/ijms26157522)
Supplement: Supplementary file 1 [file Figure_S3.pdf › Figure_S3.pdf]

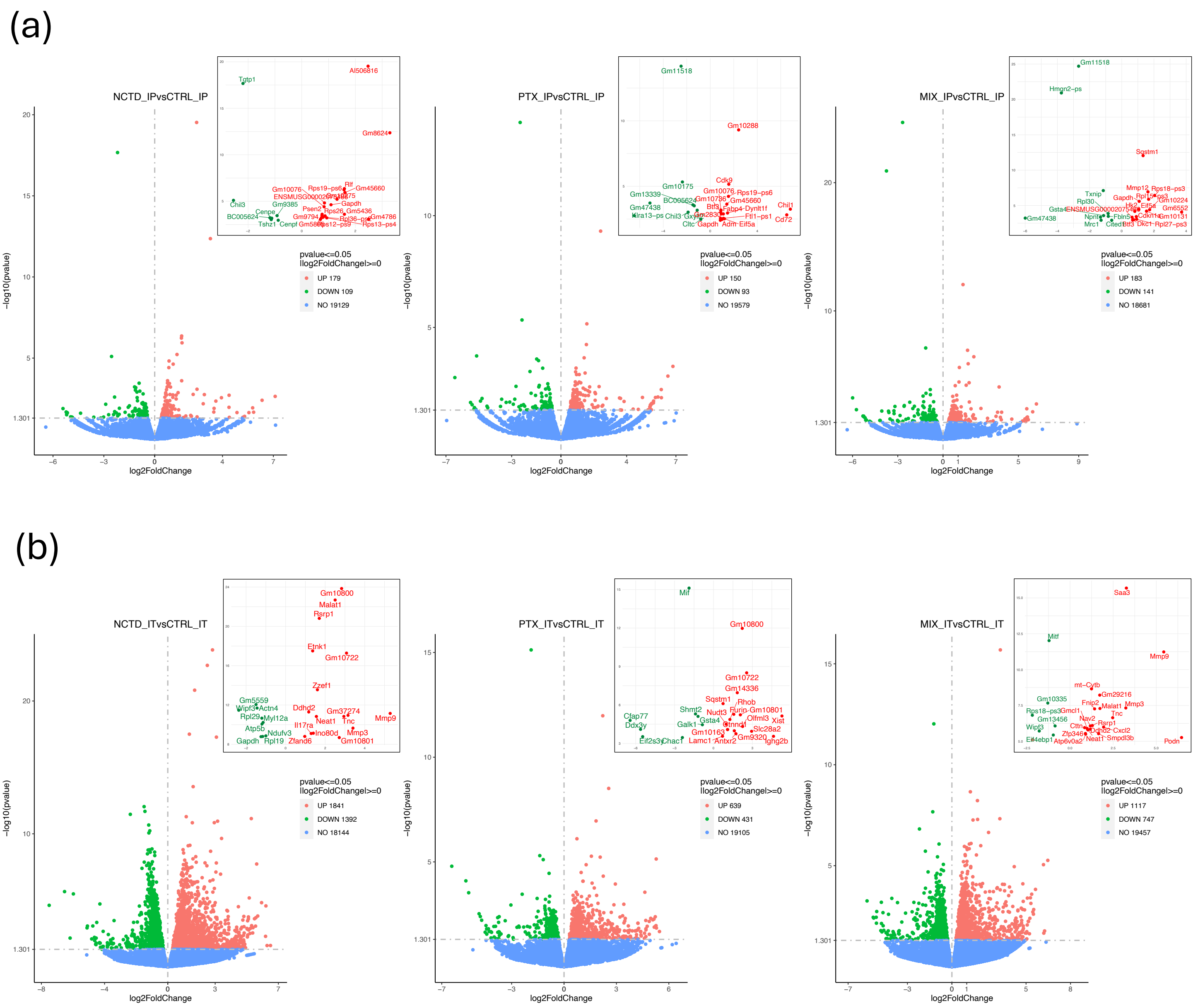

Figure S3. Volcano plots illustrating differential gene expression profiles for each treatment versus control. (a) Intraperitoneal administration groups: PTX_IP vs CONTROL_IP (243 DEGs), NCTD_IP vs CONTROL_IP (288 DEGs), and MIX_IP vs CONTROL_IP (324 DEGs). (b) Intratumoral administration groups: PTX_IT vs CONTROL_IT (1,070 DEGs), NCTD_IT vs CONTROL_IT (3,233 DEGs), and MIX_IT vs CONTROL_IT (1,864 DEGs). Each plot displays log2 fold change (x-axis) versus –log10 adjusted p-value (y-axis). Red dots represent significantly upregulated genes, green dots indicate significantly downregulated genes (adjusted $p < 0.05$, $|\log2FC| > 0$), and blue dots represent non-significant genes. In each plot, the upper right panel shows the labels of representative differentially expressed genes to facilitate visualization.
